# Detailed Profiling of 17-Hydroxygeranyllinalool Diterpene Glycosides from *Nicotiana* Species Reveals Complex Reaction Networks of Conjugation Isomers

**DOI:** 10.3390/metabo14100562

**Published:** 2024-10-20

**Authors:** Alina Ebert, Saleh Alseekh, Lucio D’Andrea, Ute Roessner, Ralph Bock, Joachim Kopka

**Affiliations:** 1 Max Planck Institute of Molecular Plant Physiology, Am Mühlenberg 1, 14476 Potsdam, Germany; 2 School of BioSciences, University of Melbourne, Parkville, VIC 3010, Australia; 3 Center for Plant Systems Biology and Biotechnology, 4000 Plovdiv, Bulgaria; 4 Research School of Biology, Australian National University, Canberra, ACT 2601, Australia

**Keywords:** plant specialised metabolism, Nicotiana, LC-MS, MS/MS, fragmentation analysis, diterpene glycosides, Nicotianosides, HGL-DTGs, plant defence

## Abstract

Background: Specialised anti-herbivory metabolites are abundant in the solanaceous genus *Nicotiana*. These metabolites include the large family of 17-hydroxygeranyllinalool diterpene glycosides (HGL-DTGs). Many HGL-DTGs occur exclusively within the *Nicotiana* genus, but information from the molecular model species *N. tabacum*, *N. benthamiana*, and the tree tobacco *N. glauca* is limited. Objectives: We studied HGL-DTG occurrence and complexity in these species with the aim of providing in-depth reference annotations and comprehensive HGL-DTG inventories. Methods: We analysed polar metabolite extracts in comparison to the previously investigated wild reference species *N. attenuata* using positive ESI(+) and negative ESI(-) mode electrospray ionisation LC-MS and MS/MS. Results: We provide annotations of 66 HGL-DTGs with in-source and MS/MS fragmentation spectra for selected HGL-DTGs with exemplary fragment interpretations of ESI(+) as well as less studied ESI(-) spectra. We assemble a potential biosynthesis pathway comparing the presence of HGL-DTGs in *N. tabacum*, *N. glauca*, and *N. benthamiana* to *N. attenuata*. Approximately one-third of HGL-DTGs are chromatographically resolved isomers of hexose, deoxyhexose, or malonate conjugates. The number of isomers is especially high for conjugates with low numbers of deoxyhexose moieties. Conclusions: We extend the number of known HGL-DTGs with a focus on *Nicotiana* model species and demonstrate that the HGL-DTG family of *N. tabacum* plants can be surprisingly complex. Our study provides an improved basis with detailed references to previous studies of wild *Nicotiana* species and enables inference of HGL-DTG pathways with required enzymes for the biosynthesis of this important family of specialised defence metabolites.

## 1. Introduction

Plants of the nightshade family (Solanaceae) have long been important models for studying specialised metabolism and their evolution. Solanaceae contain unique metabolite classes, such as different groups of alkaloids, phenylpropanoids, terpenes, and acyl-sugars [1]. The most prominent and well-investigated specialised metabolites in the genus *Nicotiana* are alkaloids, isoprenoids, flavonoids, cinnamoyl putrescines, and anthocyanins [2], but Solanaceae harbour many more classes with biological activities, such as terpenoids, sugar esters and various phenolic compounds like coumarins and lignans [3]. In addition to polar metabolites, the relative proportions of lipophilic metabolites and lipids can be substantially expanded in some *Nicotiana* species, as was revealed for the model species *N. tabacum* by metabolic diversification analyses [4]. By contrast, the tree tobacco, *N. glauca*, contains a high proportion of oxygenated specialised metabolites, acidic compounds and their derivatives [4]. *N. benthamiana* is a widely used model for transient heterologous protein expression. This species synthesises a high number of aminoglycosides, arylamines, alkaloids and organo-heterocyclic compounds [4]. This diversity illustrates that the capacity and complexity of specialised metabolism can differ substantially between species, even within a single genus.

Terpenoids were predicted to represent the most specific superclass of specialised metabolites from *N. tabacum* [4]. By contrast, O-acylglycerols and acyl-sugars were predicted as specific superclasses of *N. benthamiana* and of the wild tobacco species *N. glauca* and *N. attenuata* (coyote tobacco). The latter is known to contain the terpenoid family of 17-hydroxygeranyllinalool diterpene glycosides (HGL-DTGs). HGL-DTGs are diterpene glycosides that are abundant within the terpenoid superclass found in *Nicotiana* species, in addition to other mono-, di-, tri-, and sesquiterpenoids [3,4]. HGL-DTGs are comprised of a commonly shared diterpene aglycone, 17-hydroxygeranyllinalool (17HGL). 17HGL has glycosyl moieties attached to its two hydroxyl moieties at positions C3 and C17. The glycosyl moieties can have malonyl groups attached that further increase the diversity of this compound class. Single HGL-DTGs were already described in the 1990s for Solanaceae species [5,6,7,8]. The aglycone was isolated a decade before [9]. The HGL-DTG pathway was described mostly in the genus *Nicotiana*, foremost in *N. attenuata* [10,11,12]. The nomenclature of HGL-DTGs is divers. Some HGL-DTGs that were detected in *Nicotiana* species were called “nicotianosides”, even though HGL-DTGs also occur in other Solanaceae genera, such as *Lycium* and *Capsicum*. A key publication on HGL-DTGs chemically characterised several of these specialised compounds from *Nicotiana* species [11]. We chose this work [11] as an annotation reference for the current study that was motivated mainly by the unexpected prediction of the terpenoid superclass of specialised metabolites as a specific property of the cultivated tobacco, *N. tabacum* [4].

The biosynthesis of 17HGL requires the synthesis of geranylgeranyl diphosphate from dimethylallyl diphosphate and three isopentenyl diphosphate units by the enzyme geranylgeranyl diphosphate synthase [10]. The geranyllinalool synthase reaction creates geranyllinalool by hydroxylation at position C3 with the concomitant loss of pyrophosphate [13]. Cytochrome P450 oxidoreductases, e.g., Cyp736A304 and Cyp736A305 of *N. attenuata*, produce 17HGL by introducing the second hydroxyl group at position C17 of geranyllinalool [14]. Glucosyltransferases, i.e., uridine diphosphate glycosyltransferases (UGT), specifically UGT74P3 and potentially UGT74P5, add hexose moieties to the hydroxyl moieties of 17HGL. Rhamnosyltransferase UGT91T1 is thought to add deoxyhexose moieties to the hexose decorations [12]. NMR analyses of HGL-DTGs from *N. attenuata* demonstrated that the hexose moieties are glucose and all deoxyhexoses are rhamnose [11]. The glucose-to-glucose glycosidic bonds detected were exclusively (2->1) linked, whereas the glucose-to-rhamnose bonds had (4->1) linkage [11]. Older studies described the presence of glucose-to-rhamnose (6->1) linkages in penta-glycosylated HGL-DTGs from *N. tabacum* [6,7]. Analysis of the stereoisomerism of the sugars, lyciumoside I, II and IV, as well as attenoside and two penta-glycosylated HGL-DTGs, demonstrated the presence of β-D-glucopyranosyl/β-D-glucopyranoside moieties [5,6,8,10] and α-L-rhamnopyranosyl moieties [6,8,10]. Other HGL-DTGs of *Nicotiana* species have not been examined in terms of their sugar stereoisomerism. A malonyl-group can be attached to position 6 of a hexose (glucose) moiety by malonyltransferase 1 [11,15]. In our present MS-based study, we will address glycosides as hexoses or deoxyhexoses unless demonstrated otherwise.

Even though the exact mechanism of action is still unclear, HGL-DTGs have a defence function against herbivory by insect larvae, e.g., of the tobacco hornworm [10,16,17] and the tobacco budworm [7]. HGL-DTGs are inducible in shoot tissues upon wounding and application of oral secretion from *Manduca sexta* [10]. Silencing of glycosyltransferases acting on 17HGL increases toxicity to larvae, likely through accumulation of HGL-DTG intermediates, increased total HGL-DTG pools, and/or enhanced 17HGL release upon feeding [12]. Notably, the aglycone 17HGL is auto-toxic to *N. attenuata,* and sugar conjugation was proposed as a likely mode of action to avoid auto-toxicity [12].

Apart from the differences in their specialised metabolism, the *Nicotiana* species selected for this study have diverse geographical origins, with *N. tabacum* and *N. glauca* originating from South America, *N. attenuata* from North America and *N. benthamiana* from Australia. Furthermore, the selected species include different stages of ploidy, with the diploid species *N. glauca* and *N. attenuata*, as well as the allotetraploid species *N. benthamiana* and *N. tabacum* [18,19]. The cultivation of *N. tabacum* and *N. benthamiana* might have impacted their chemical-ecological properties, in addition to their origin by allopolyploidisation. *N. tabacum* was cultivated by humans for use as a drug, and its cultivation spread over South and Central America already in pre-Columbian times and, since then, all over the world [20]. Hence, *N. tabacum* cultivars can vary substantially in their metabolic properties [20]. Furthermore, *N. tabacum* and cell lines thereof have become widely used models to study the molecular processes of plants. The laboratory strains of the other model species, *N. benthamiana*, are quite homogenous across the world and probably originate from a single seed batch collected in the 1930s in the Northern Territory (Australia) [21]. *N. glauca* has been used as an ornamental plant by humans and has become invasive on all continents in dry temperate and tropical regions [22].

Despite recent advances, we still lack detailed insight into the complexity of HGL-DTG synthesis in *N. tabacum* and other molecular model species of the genus. Deeper insights into the HGL-DTG inventories of model species are needed to investigate the role of this compound family in plant stress responses and to elucidate their mode of action against herbivorous insects. Our objectives are to fill the knowledge gaps of HGL-DTG complexity in *N. tabacum*, *N. benthamiana* and *N. glauca* and to infer the presence of potential biosynthesis paths of glycosylation and malonyl transfer in these key model species. We carefully place our data in the context of previously described HGL-DTGs and their occurrence in wild species of the *Nicotiana* genus. Based on the new information we have obtained, we propose a concise reaction pathway that summarises the current knowledge of HGL-DTG synthesis in the *Nicotiana* genus.

## 2. Materials and Methods

### 2.1. Plant Material

*N. tabacum* cv. Samsun NN (SNN) and *N. glauca* were grown together in one experiment (Exp 1), and a second batch of *N. tabacum* plants was grown together with *N. benthamiana* in a second experiment (Exp 2, Figure S1). The plant lines of this study are long-term laboratory cultivars that are in-house propagated marker and reporter lines with either a hygromycine (*N. tabacum*) or a kanamycine resistance cassette and a *YFP* reporter gene (*N. glauca* and *N. benthamiana*) in their nuclear genomes [23,24,25]. Plants were grown from seeds germinated on a synthetic medium and then transferred to soil (Exp 1) or initially raised in a sterile culture and then transferred to soil and grown to maturity (Exp 2) in a growth chamber in a 16 h light/8 h dark diurnal rhythm. The day conditions were 22 °C, 75% humidity and a light intensity of 350 µmol photons m^−2^ s^−1^; the night conditions were 18 °C and 70% humidity. The aboveground biomass, with the exception of senescent or damaged leaves and the lower part of the stem, was harvested at a developmental stage when the plants had approximately 10 fully expanded leaves. The plant material was directly frozen in liquid nitrogen and stored until processing at −80 °C. Samples were ground with mortar and pestle under liquid nitrogen. *N. attenuata* control plants were grown in tissue culture and directly frozen in liquid nitrogen at harvest.

### 2.2. Metabolite Preparation

Frozen samples of 50 mg (±3 mg) fresh weight (FW) were extracted in a mixture of 350:400:200 (*v*/*v*/*v*) methanol:water:chloroform. A 300 µL aliquot was transferred to new plastic tubes, dried in a vacuum concentrator at room temperature overnight and stored at −20 °C before measurement. The extraction procedure consisted of the addition of pre-cooled (−20 °C) methanol, including the internal standard to the frozen plant material, heating of the extracts at 70 °C for 15 min while shaking, the addition of chloroform, heating at 37 °C for 5 min while shaking, the addition of water and final centrifugation for 5 min at 14,000 rpm at room temperature in a benchtop centrifuge. Dried extracts of each replicate were re-dissolved in 200 µL water before routine LC-MS measurements. Extracts for LC-MS/MS measurements were re-dissolved in 50 µL 50% (*v*/*v*) methanol:water mixture; for the latter, multiple random replicates of the same species were pooled to obtain a representative sample.

### 2.3. LC-MS Analysis

Reversed-phase measurements were performed with a Waters Acquity UPLC system and a C_18_ column (100 mm × 2.1 mm containing 1.7 μm diameter particles, Waters) connected to an Exactive Orbitrap-type MS or Exactive Orbitrap-focus (Thermo Fisher Scientific, Waltham, MA, USA) for MS/MS measurements. The injection volume was 3 µL. The 20 min chromatography was executed with a stable flow of 0.4 mL/min. Solvents were A, water, and B, acetonitrile, both containing 0.1% formic acid. Column temperature was constantly at 40 °C. Starting with 99% A for 1 min, a linear gradient was set to 60% A until 11 min, and from then on to 30% A until 13 min. Finally, a linear gradient flush up to 99% B was programmed until 15 min and held for 1 min before returning to 99% A within 1 min. 99% A was held for the remaining chromatography time to equilibrate to start settings. For detection, molecules were ionised by electrospray ionisation. Full mass range spectra were acquired in ESI(+) and ESI(-) ionisation modes ranging from mass to charge ratio (*m*/*z*) 100 to 1500 during the 0–19 min chromatography period. MS/MS spectra of metabolites were acquired in ESI(+) and ESI(-) mode at a collision energy of 25 eV by data-dependent tandem mass spectrometry (ddMS^2^). FT resolution was 25,000 for MS and 7500 for ddMS^2^. See [26] for more detailed LC, MS and MS/MS settings.

### 2.4. Metabolomic Data Processing

LC raw files were processed by Refiner MS software from Genedata Expressionist^®^ version 14.0.3 (http://www.genedata.com, accessed on 18 September 2022) [26]. The chromatography data processing output contained summed cluster abundances (“clustersum”), adding all detected isotopologue abundances. The abundance data were normalised to the abundance of the internal standard Crocin2 (CAS Number 55750-84-0, Sigma-Aldrich/Merck: PHL80392, manufacturer: Phytolab GmbH & Co.KG, Vestenbergsgreuth, Germany). The averaged background of the non-sample and water-only controls was subtracted from the initial abundances. Data were normalised to the amount of plant FW and further to the mean value of the respective mass feature of *N. tabacum* as the reference species used consistently across all runs and extractions (thus providing comparability between all analytical runs and experiments). The normalisation of the abundance levels of the internal standard accounts for potential variations due to matrix effects between samples and for manual handling errors during extraction. Crocin2 (also “Tricrocin” or “Crocetingentiobiosylglucosyl ester”) was chosen as an internal standard because it resembles the molecular properties of 17-hydroxygeranyllinalool diterpene glycosides (HGL-DTGs). It was the chemically closest of all commercially available standard compounds and had a terpenoid backbone of 20 carbon atoms as well. It harbours all-glucose glycosylations at both ends of the aglycone and resembles with its tri-glycosylation the average of the di- to penta-glycosylated HGL-DTGs.

### 2.5. HGL-DTG Annotation

For extant HGL-DTG annotation and references, see [11]. The annotation process in this study consisted of the following steps: confirming the presence of the aglycone in ESI(+) measurements, identifying the [M+NH_4_]^+^ adduct in ESI(+) mode, manually identifying within chromatogram files the number of isomers separated by our chromatography and comparing *m*/*z* and retention times, i.e., the analysis of the chromatographic retention sequence, to described HGL-DTGs in [11] (cf. Supplementary Tables S2 and S6). In ESI(+) mode, HGL-DTG mass spectra typically exhibited the aglycone mass fragment of *m*/*z* 271.24 without all sugar moieties and hydroxyl groups, as described before [11]. The DTGs were annotated by the presence of the aglycone mass fragment *m*/*z* 271.24 in ESI(+) mode co-occurring at the same timepoint with a [M+NH_4_]^+^ adduct using extracted ion chromatograms (EIC). The presence of the aglycone mass was additionally verified by independently measured MS/MS spectra, if obtained by the automated peak picking ddMS^2^. If present, the [M-H]^−^ mass feature of the ESI(-) mode was used for relative quantification due to only marginal fragmentation in this mode. The [M+H]^+^ ion was barely detectable by ESI(+) and was only available in rare cases. Use of the adduct mass feature [M+NH_4_]^+^ for quantification was assumed to be less accurate than the [M-H]^−^ as the ESI(+) mode caused extensive in-source fragmentation of HGL-DTGs.

For pathway visualisation and mapping, the software VANTED version 2.8.8 (https://www.cls.uni-konstanz.de/software/vanted/, accessed on 26 July 2021) was employed [27]. Structures were obtained by the ChemSketch software version 2021.2.1 (https://www.acdlabs.com/resources/free-chemistry-software-apps/chemsketch-freeware/, accessed on 9 October 2023). Chromatographic data were visualised by the Xcalibur Freestyle software version 1.8.63.0.

## 3. Results

We performed LC-MS(/MS) analyses to investigate the 17-hydroxygeranyllinalool diterpene glycoside (HGL-DTG)-related metabolic capacity of selected *Nicotiana* species. The aim of this study was to gain a detailed overview of existing HGL-DTG species and their isomer numbers. For this purpose, fragmentation and fragment interpretation in ESI(+) and ESI(-) ionisation modes were investigated. A complete list of detectable HGL-DTGs in the species was compiled and put into a pathway context.

### 3.1. Detection of HGL-DTGs in Four Nicotiana Species via Presence of the Aglycone

**Figure 1:**
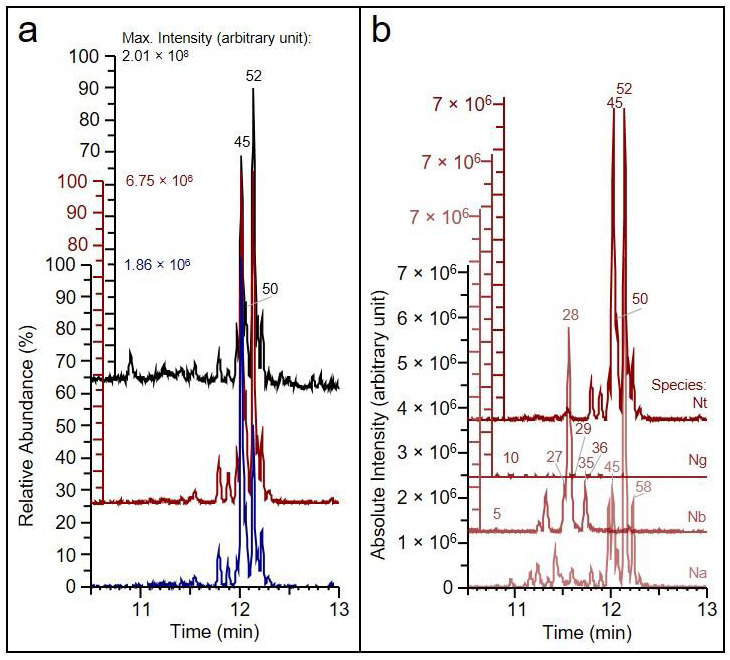
HGL-DTG analyses of selected *Nicotiana* species. (**a**) ESI(+) mode total ion chromatogram (black) of *N. tabacum* and extracted ion chromatograms of the aglycone fragments after elimination of all OH groups (C_20_H_31_+, *m*/*z* 271.2420, red) or with one OH group remaining (C_20_H_33_O+, *m*/*z* 289.2526, blue). (**b**) Extracted ion chromatograms of the aglycone fragment C_20_H_31_+, *m*/*z* 271.2420 (red), of *N. tabacum* (Nt), *N. glauca* (Ng), *N. benthamiana* (Nb) and *N. attenuata* (Na). The presence of the aglycone fragments supports the annotation of HGL-DTGs. The mass tolerance of single ion chromatogram masses was ±1 ppm. Extracted and total ion chromatograms of HGL-DTGs at 10.5 to 13.0 min chromatography time were created with the Xcalibur Freestyle software version 1.8.63.0. The y-axes are displayed with an offset to visualise the different maximum-scaled abundances (%) or absolute intensities (arbitrary units). Chromatogram traces are displayed without offset. The three most abundant HGL-DTGs of each chromatogram, as well as the HGL-DTGs analysed in Section 3.2, are labelled in the figure with their order number (see Section 3.3).

**Figure 2:**
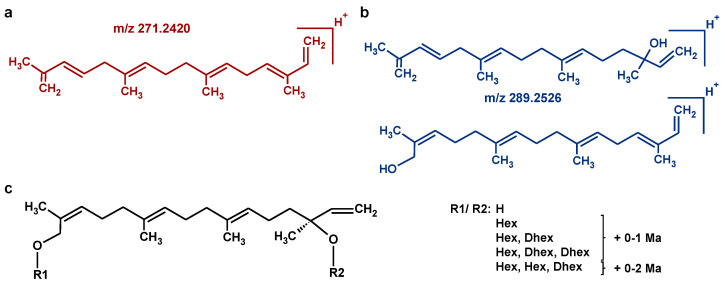
The general structure of HGL-DTGs and hypothetical structures of aglycone fragments. (**a**) Aglycone fragment without OH groups (C_20_H_31_+, *m*/*z* 271.2420, red). (**b**) Two potential isomers of aglycone fragments with one OH group attached (C_20_H_33_O+, *m*/*z* 289.2526, blue). (**c**) Generalised structure of HGL-DTGs where potential substituents are H atoms or diverse sugar moieties with or without attached malonyl groups. The presented possibilities of R1 and R2 are based on previously observed and published structures. Structures were modified from drawings made with the ChemSketch software version 2021.2.1.

The main metabolite class eluting between chromatography time 11.5 and 12.5 min of our analyses is the class of HGL-DTGs (Figure 1), as demonstrated exemplarily by extracted ion chromatograms *m*/*z* 271.2420 and *m*/*z* 289.2526 of the HGL-aglycone compared to the respective total ion chromatogram (Figure 1a) and by extracted ion chromatograms at *m*/*z* 271.2420 of the *Nicotiana* species from this study (Figure 1b). The aglycone fragments detectable by in-source fragmentation either bear no hydroxyl group or only one (Figure 2a,b). A potential aglycone fragment with both hydroxyl groups attached, i.e., with a hydrogen atom at R1 and R2 (Figure 2c), is not detectable. Both aglycone fragments, *m*/*z* 271.24 and 289.25, show a MS/MS fragmentation pattern typical of terpene structures (Figure S2). The aglycone fragment *m*/*z* 271.24, representing the elimination of an additional hydroxyl group compared to *m*/*z* 289.25, is likely a sequential product ion after the elimination of the first substituent at R1 or R2. Fragment *m*/*z* 289.25 is likely a mixture of two possible isomers, either with a hydroxyl group remaining at position R1 or at R2, where the remaining hydroxyl group is likely the result of an elimination reaction from the substituent sugar moiety, or may be part of the initial HGL-DTG structure. HGL-DTG diversity and abundance are quite distinctive of the different selected *Nicotiana* species (Figure 1b). Among the compared species, *N. tabacum* has the highest abundances of individual HGL-DTGs, whereas *N. glauca* appears to synthesise only low amounts of this metabolite class.

### 3.2. Characteristic Fragmentation Reactions of HGL-DTGs

To analyse the characteristic mass spectral fragmentation of HGL-DTGs, we first investigated exemplarily the HGL-DTG 862.4 e, i.e., nicotianoside Ic. Due to the diverse nomenclature of HGL-DTGs, we chose to name nicotianosides systematically by molecular mass at 0.1 amu resolution and elution sequence indicated by lowercase letters and additionally provide previous names at first mention for clarity.

We compared HGL-DTG 862.4 e (Figure S3, Table S1) to the fragmentation reactions of isomeric HGL-DTG 862.4 d, i.e., nicotianoside Ib [11]. Five abundant fragments were previously reported by a ESI(+) mode in-source and MS/MS analysis at collision energy 20 eV of the ammonium adduct of HGL-DTG 862.4 d [11]. We detected and confirmed m/z 271.24 ([Aglyc-2H_2_O+H]^+^), 417.30 ([Aglyc+Rha-3H_2_O+H]^+^) and 880.45 ([M+NH_4_]^+^) by MS/MS analysis at collision energy 25 eV using the ammonium adduct of HGL-DTG 862.4 e (Figure S3c, Table S1). The other two previously reported mass fragments *m*/*z* 537.31 ([Aglyc+Glc+Ma-2H_2_O+H]^+^) and 683.36 ([Aglyc+Rha+Glc+Ma-4H_2_O+H]^+^) were detectable by our in-source fragmentation analysis of HGL-DTG 862.4 e (Figure S3a, Table S1), but not by MS/MS. This difference is likely due to the changed collision energy and instrument settings or the difference in the isomeric HGL-DTG structures. Comparison to the previously reported eight abundant fragments from an ESI(+) mode MS/MS spectrum of the hydrogen adduct of HGL-DTG 862.4 d [11] confirmed *m*/*z* 271.24, 289.25 ([Aglyc-H_2_O+H]^+^), 395.12 ([Rha+Glc+Ma-3H_2_O+H]^+^), 417.30 and 597.36 ([Aglyc+Rha+Glc-3H_2_O+H]^+^) among the ten most abundant fragments from our in-source analysis of the ammonium adduct of HGL-DTG 862.4 e (Figure S3a). Our MS/MS analysis of HGL-DTG 862.4 e (Figure S3c) contains only *m*/*z* 271.24, 289.25 and 395.12 under the top ten most abundant fragments. *M*/*z* 417.30, 557.17 ([Rha+Glc+Glc+Ma-4H2O+H]+) and 597.36 are present in our MS/MS spectra at low abundance. The hydrogen adduct, *m*/*z* 863.43 ([M+H]^+^), is naturally not present in our MS/MS analyses of the ammonium adduct of *m*/*z* 880.45 ([M+NH_4_]^+^). All other fragments of HGL-DTG 862.4 e that had previously been reported in the supplemental data of [11] were detected by our MS/MS or in-source analyses and confirmed the identity of our HGL-DTG annotation with the previous report, together with their presence within co-analysed nicotianoside preparations from *N. attenuata*. Characteristic losses and fragment masses that can be observed by ESI(+) mode analysis contain single-sugars moieties, sugar chains with and without an attached malonyl group, as well as the aglycone moiety and the aglycone in combination with sugars and malonyl groups (Table S1).

Our study was consistent with previous observations [11] of fragments *m*/*z* 417.30 ([Aglyc+Rha-3H_2_O+H]^+^) and 435.31 ([Aglyc+Rha-2H_2_O+H]^+^) (Tables S1 and S2) that originate from rhamnosyl moieties of the selected structurally characterised HGL-DTGs. These observations indicate intramolecular rearrangements after ionisation. NMR studies confirmed that HGL-DTG 862.4 d and e (nicotianosides Ib and Ic) do not contain rhamnose that is directly attached to the aglycone backbone [11]. Glucose is the first sugar conjugated both at R1 and R2 of the aglycone. Hence, mass spectral fragmentation analyses of HGL-DTGs are not sufficient to unambiguously assign sugars linked to the aglycone or determine the sequence of sugar chains.

In addition to confirming ESI(+) mode mass spectral properties, we investigated mass spectra obtained by ESI(-) mode ionisation. These mass spectra are less informative for structure predictions as only a few fragmentation reactions occurred. Malonyl losses from the exemplary HGL-DTGs were detectable, as well as losses of part of the conjugated sugar moieties. The aglycone fragments were not detectable (Figure S3b,d, Table S1). Fragments of the conjugated sugar structures are rarely detectable. Negative mode analysis generates stable [M-H]^−^ ionisation products of HGL-DTGs. At identical collision energies, [M-H]^−^ is still detectable at high abundance, whereas the ESI(+) mode ionisation product [M+H]^+^ is always lowly abundant or even undetectable. Due to fewer fragmentation reactions in ESI(-) mode, the [M-H]^−^ ionisation product efficiently confirms the molecular mass abundance of HGL-DTGs that have been annotated from ESI(+) mode mass spectra.

**Figure 3:**
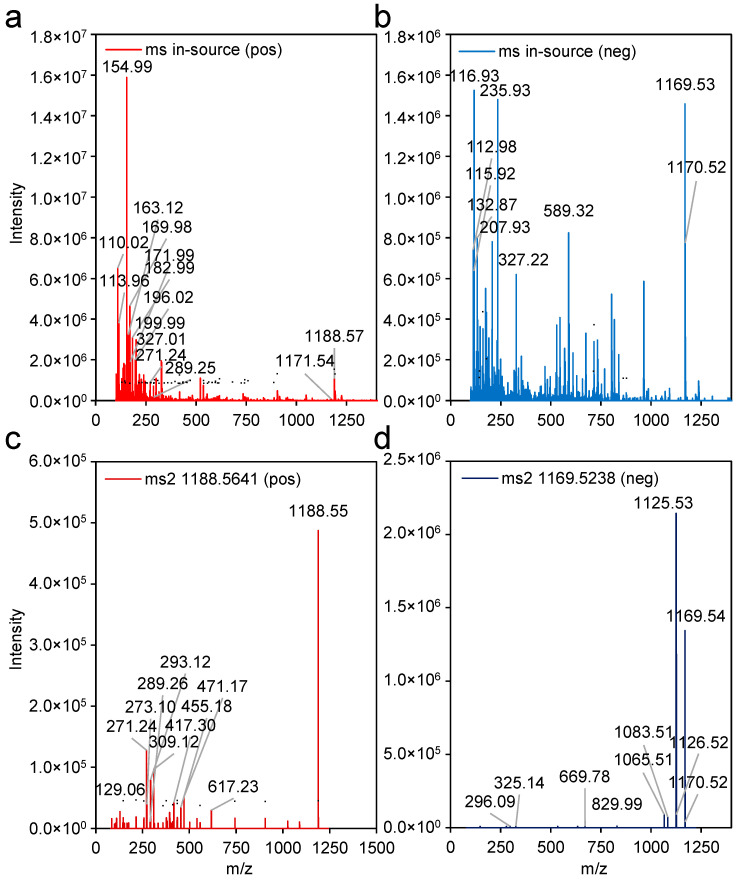
Exemplary fragmentation analysis of HGL-DTG 1170.5 b, i.e., DTG 1188′′, by ESI(+) mode ionisation (**a**,**c**, red) and ESI(-) mode ionisation (**b**,**d**, blue) mass spectrometry. Fragment ions of in-source fragments (**a**,**b**) and from MS/MS (ms2) fragmentation experiments at collision energy = 25 eV (**c**,**d**) are compared. Data are from the same sample of *N. tabacum* cv. Samsun NN. Mass spectra were extracted at retention times of 10.88 min (ESI(+) mode) and 10.86 min (ESI(-) mode), respectively. The top ten most abundant fragments and the most relevant fragments are indicated by mass-to-charge ratios (*m*/*z*). Further annotated fragments (Table S2) are labelled by dots. Reported intensities have arbitrary units.

The confirmed fragmentation characteristics of the structurally well-characterised HGL-DTG isomers 862.4 d and e enabled the mass spectra annotation process of all observed HGL-DTGs and newly reported isomers. We chose HGL-DTG 1170.5 b (DTG 1188′′) to exemplify the process (Figure 3). HGL-DTG 1170.5 b harbours five sugar moieties and one malonyl group, has medium to low abundance in samples of *N. tabacum* and has not yet been described in detail. Due to its relatively low abundance and structure complexity, in-source fragmentation from both ESI(+) and ESI(-) mode analyses are more complex and potentially compromised by chromatographic background noise or co-eluting compounds (Figure 3a,b). MS/MS fragmentation is required to clarify and demonstrate expected fragmentation reactions caused by sugar and malonyl losses (Table S2). MS/MS fragmentation of the adduct *m*/*z* 1188.56 ([M+NH_4_]^+^) in ESI(+) mode indicated the presence of at least 2 hexoses (Hex), 2 deoxyhexoses (DHex), and 1 malonylation (Ma) next to the presence of the 2 characteristic aglycone fragments (Table S2). The fragments, including the aglycone, contained between 1 and 4 sugar moieties that did not contain more than 2 Hex or 2 DHex moieties. Fragments without the aglycone demonstrated the presence of only one malonylation and not more than 4 sugar moieties and 2 Hex or 2 DHex with the respective water molecule eliminations. Only in-source fragments had 5 attached sugar moieties. MS/MS analyses in ESI(-) mode confirmed conjugation of 3 Hex and 2 DHex to the aglycone. The distribution of conjugations between R1 and R2 of HGL, the linkage between Hex, DHex, and Ma, as well as the identity of Hex (i.e., glucose or DHex, e.g., rhamnose), was not elucidated.

### 3.3. Comprehensive Annotation of HGL-DTGs from N. tabacum, N. glauca and N. benthamiana with Reference to N. attenuata

We detected 66 HGL-DTGs, including isomers of 22 predicted molecular formulas (Table 1). By detailed analysis of these isomers that were resolved by chromatographic retention, we increased the number of observable HGL-DTGs approximately threefold. To obtain a comprehensive overview of the synthesis capacity of the investigated *Nicotiana* species and to understand the enzymatic requirements of glycosyl- and malonyl-transferases that may explain the observed diversity of HGL-DTGs, we included lowly abundant isomers as long as these met our annotation criteria (see Section 2.5). We collected the highest possible diversity of HGL-DTGs and included HGL-DTGs of low abundance because our study investigated this metabolite family at optimal growth conditions. We take into account that HGL-DTGs may be lowly abundant in the non-induced state but may accumulate upon exposure to biotic or non-biotic stresses when their biological defence functions are required.

We report putative sugar decorations of the HGL-DTGs as Hex or DHex (Table 1) in agreement with the characteristics and limitations of the LC-MS method that we used. Mass spectrometry typically does not differentiate between sugar isomers and different linkages within a molecule. HGL-DTGs were annotated in agreement with previous LC-MS and NMR reports, e.g., [11], and classified by a number of hexoses followed by deoxyhexoses and malonylations, respectively (Table 1). We annotated 50 previously reported HGL-DTGs and 16 additional isomers of known HGL-DTGs grouped by their predicted molecular formulas. Several of these HGL-DTG isomers may have been overlooked due to their low abundance, species-specific occurrence, and/or the analytical performance differences of chromatographic separations. In summary, we report HGL-DTGs that contain 2–5 sugar moieties either comprising Hex or a mixture of Hex and DHex conjugations. The numbers of Hex, DHex or Ma residues do not exceed 3, e.g., HGL-DTG 1256.5 a and b containing 3 Hex, 2 DHex and 2 Ma decorations, or HGL-DTG 1154.5 with 2 Hex, 3 DHex and 1 Ma residue (Table 1). We confirm the presence of HGL-DTG 630.4, i.e., lyciumoside I, which contains only 2 Hex moieties and may be at the basis of the HGL-DTG biosynthesis pathway. An in-depth search for metabolic precursors with only 1 Hex residue or 1 DHex failed to demonstrate their presence.

We carefully matched our HGL-DTG annotations to previous reports from other Solanaceae species and to our own measurements of reference samples from the previously studied species *N. attenuata*. Only 27 HGL-DTGs, i.e., ~41% of the described HGL-DTGs, have a known structure (Table 2). For those, glucose and rhamnose were identified as constituent Hex and DHex moieties, respectively, and for most, the linkages of glycosidic bonds are known (Table 2). Our analyses confirmed all previously described HGL-DTGs from *N. attenuata* that were also present in the species studied here. Most of the newly described HGL-DTG isomers were absent from the *N. attenuata* reference samples and appear to be characteristic of the selected additional *Nicotiana* species. Structures of HGL-DTG 1068.5 and 1084.5 (a, b, or c) have been described after isolation from *N. tabacum* material [6]. Due to incomparable chromatography between the previous [6] and this study, we cannot exactly match these structures to our assignment of HGL-DTG isomers. To comply with good annotation praxis, we provide a general metabolic annotation quality level [28] and report HGL-DTG-specific annotation classes of this study (Table 2).

To provide a basis for future comparative LC-MS studies of HGL-DTG metabolism, we provide detailed information on retention time and retention time ranges from our experiments, together with molecular ions for potential quantification, and characterise typical m/z errors of measurement. We analysed [M+NH_4_]^+^ adducts and [M-H]^−^ from ESI(+) and ESI(-) mode analyses, respectively. Availabilities of MS/MS spectra are indicated (Table S3). A representative MS/MS spectrum of each isomer group is reported with the ten most abundant MS/MS product ions of the molecular ions [M+NH_4_]^+^ and [M-H]^−^, if available from ddMS^2^ analysis (Table S4).

### 3.4. Occurrence of N. tabacum, N. glauca, N. benthamiana HGL-DTGs in the Analysed Nicotiana Species

We also compared different cultivation regimes for *N. tabacum* plants. In one experiment, seeds were germinated in vitro on a synthetic medium and then transferred to soil. In another experiment, plantlets were pre-grown in vitro and then transferred to soil. These growing procedures did not substantially impact the HGL-DTG composition. *N. tabacum* plants from tissue culture (Exp 2) contain about as many HGL-DTGs at similar developmental stages after transfer to soil. 45 (Exp 2) and 47 (Exp1) HGL-DTGs were detected in at least one plant of the respective experiment, with an intersection of 35 common HGL-DTGs, i.e., 78% (Exp 2) and 74% (Exp 1).

**Figure 4:**
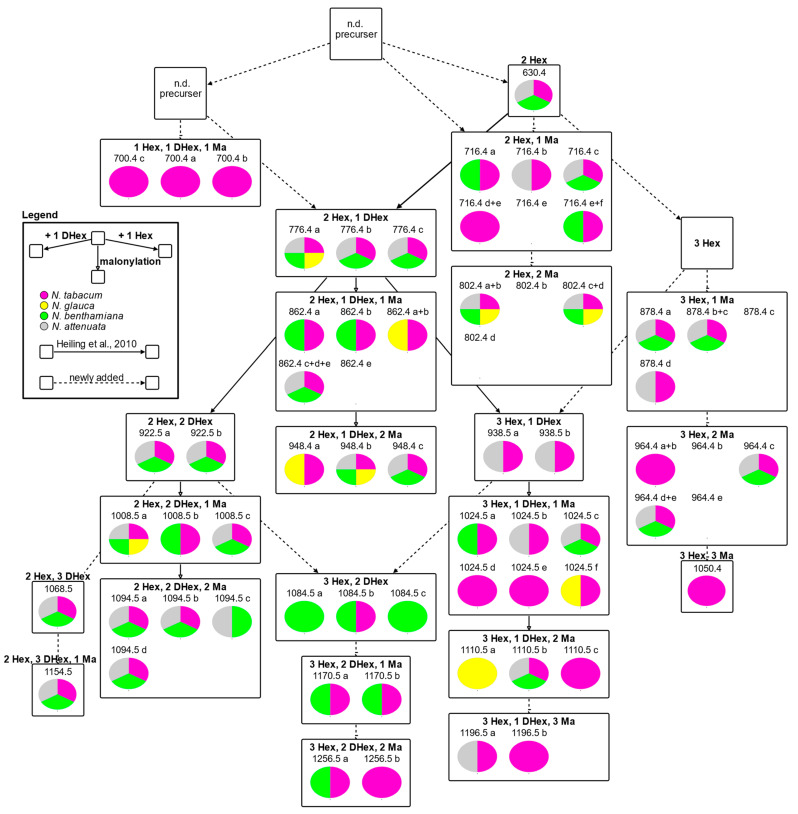
Potential biosynthesis pathways of HGL-DTGs present in *N. tabacum* (pink), *N. glauca* (yellow), and *N. benthamiana* (green) compared to their presence in *N. attenuata* (grey). The presence of individual HGL-DTGs in the four *Nicotiana* species is represented by pie charts. Presence was scored if an isomer was detected in at least 50% of the samples from a species and in at least one experiment (*n* = 4–12 plants). Automated data processing was manually curated to correctly annotate isomers. Previously proposed reactions [10] are represented by solid arrows, and newly proposed reactions are represented by arrows with dotted lines. Boxes indicate isomer groups. The HGL-DTG name is composed of molecular mass and a lowercase letter indicating the elution sequence within a group of isomers (a–f). Glycosylation and malonylation reactions are indicated by the direction of the arrows, as shown in the inset (left orientation: addition of a deoxyhexose; right orientation: addition of a hexose; downward arrow: malonylation). The graph was created with the VANTED software version 2.8.8. N.d.: not detected.

We mapped HGL-DTG occurrences onto a schematic pathway of sequential HGL-DTG decorations (Figure 4). This scheme is based on previously proposed reaction paths [10]. Because the sequence of conjugation reactions is currently elusive, we consider multiple potential precursors of the different groups of HGL-DTG isomers. For the comparison of HGL-DTG occurrence, we considered HGL-DTGs that were robustly present in 50% of all samples from a single species in at least one of the experiments.

One of the newly proposed reactions relates to HGL-DTG 700.4 isomers with predicted 1 Hex, 1 DHex, and 1 Ma decorations. HGL-DTG 630.4 (2 Hex) cannot be the direct precursor of HGL-DTG 700.4 a, b, and c, but we failed to detect a mono-glycosylated precursor molecule. HGL-DTG 716.4 (2 Hex, 1 Ma) is a group with an exceptionally high number of isomers and highly diverse chromatographic retention times ranging from 10.62 min (HGL-DTG 716.4 a) to 12.07 min (HGL-DTG 716.4 c) and 12.28 (HGL-DTG 716.4 e) (Table S3). Such differences in chromatographic retention imply variation in structural polarity among this group of isomers. Three possible explanations should be considered. (1) HGL-DTG 630.4 (2 Hex) as a potential precursor may receive malonylations at multiple positions of the Hex residues. Heiling et al. [11], however, argue, based on their NMR observation, that malonyl groups are exclusively attached to position 6 of Hex residues. Other Hex positions and rhamnose appear not to be malonylated. Consequently, only 2 HGL-DTG 716.4 isomers should exist. (2) HGL-DTG 630.4 (2 Hex) may have other hexoses than glucose attached. (3) A chain of two Hex residues may be attached at R1 or R2. We did not detect isomers of HGL-DTG 630.4 or mono-glucosylated HGLs that would support options (2) or (3). To accommodate these potential reaction paths, we suggest an alternative precursor to HGL-DTG 630.4 for the biosynthesis of a subset of HGL-DTG 716.4 isomers.

The number of detected highly decorated isomers does not reflect the number of possible conjugation combinations and indicated enzymatic constraints of HGL-DTG biosynthesis. HGL-DTGs with five sugar moieties have low numbers of isomers. We suggest that position-specific paths for the synthesis of one or a few highly glycosylated HGL-DTGs exist but cannot rule out that the respective isomers may be more difficult to resolve.

Our study demonstrates that *N. tabacum* cv. Samsun NN synthesises the largest variety of HGL-DTGs among the species investigated and exclusively accumulates HGL-DTG 700.4 isomers and HGL-DTG 1050.4. *N. benthamiana* synthesises a high number of HGL-DTGs that mostly match those of *N. tabacum* but also synthesises unique isomers in the highly decorated branches (Figure 4). *N. glauca* accumulates a few HGL-DTGs, including a specific, highly decorated isomer (HGL-DTG 1110.5 a). *N. glauca* appears to lack most HGL-DTG precursors and intermediates. Likely, these precursors and intermediates are too low in abundance to be detectable.

## 4. Discussion

Based on plastome phylogenetic analysis, the allotetraploid *N. tabacum* separated on the maternal side ~6 million years ago from the lineages of the diploid *N. glauca* and the allotetraploid *N. benthamiana* (maternally) and ~10 million years ago from the diploid *N. attenuata* [30]. On the paternal side, *N. tabacum* separated ~11 million years ago from the other three species lineages [30]. The ability to produce HGL-DTGs seems to be conserved in the genus, considering that the species investigated in this study stem from widely different geographic locations (the Americas and Australia) and are distantly related inside the *Nicotiana* genus. They also represent different ploidy levels. Based on the data obtained in our present study on the occurrence of the different HGL-DTGs, most of the species are able to synthesise members of all branches in the pathway, with the exception of *N. glauca,* which appears to lack HGL-DTGs with 2 Hex + 3 DHex, 3 Hex or 3 Hex + 2 DHex. Furthermore, only *N. tabacum* accumulated HGL-DTGs with 1 Hex + 1 DHex + 1 Ma. The similarity of HGL-DTG synthesis capacity is especially striking for *N. benthamiana,* which produces nearly the complete HGL-DTG range of *N. tabacum*, the species producing most HGL-DTG isomers detected in our study. The similarity between these two allopolyploid species could be due to an ancestor of the Sylvestres section being a shared progenitor species of both *N. tabacum* and *N. benthamiana* [30,31,32].

The *N. tabacum* Samsun NN cultivar used in this study seems to be different in the synthesis activity of HGL-DTGs compared to *N. tabacum* var. Maryland Mammoth [11]. The lack of HGL-DTG accumulation in the latter was also reported for one of the diploid progenitor species, *N. sylvestris* [11]. By contrast, we detected a complex profile of different HGL-DTGs that is more similar to the reported profile of the other diploid progenitor species, *N. tomentosiformis* [11]. In this regard, the Samsun NN cultivar seems to be similar to the *N. tabacum* var. Xanthi nn cultivar which has been reported to contain a complex HGL-DTG profile with di- to tetra-glycosylated members of the HGL-DTG family [33]. It seems possible that, through their long cultivation by humans, different *N. tabacum* cultivars might have been selected for differential metabolic phenotypes. Since specialised metabolism determines important traits that are commonly selected for plant domestication (including plant defence and accumulation of aroma-related compounds), it seems conceivable that the HGL-DTG spectrum and accumulation levels have been affected by human selection. The cultivars Samsun and Xanthi have been cultivated in regions of today’s Greece and Turkey and were marketed as “Turkish tobacco”. Thus, their breeding in the same area could explain the similarity of the HGL-DTG profiles of the two varieties, as well as the markedly different profile of the North American cultivar Maryland Mammoth. Another phenotypic trait that differs between these cultivars is flowering, where, due to a mutation, Maryland Mammoth does not flower under long-day conditions, in contrast to most other *N. tabacum* cultivars [34].

In the diploid species *N. glauca*, the pathway appears to be least active. However, it cannot be excluded that *N. glauca* would synthesise more HGL-DTGs upon herbivory, given that HGL-DTG accumulation can be inducible upon attack by pest insects [10,11,35]. As the only perennial and woody species in our study, next to the herbaceous and mostly annual species, *N. glauca* may have a different trade-off strategy in balancing growth and defence [36], for example, by investing more in rapid growth at early developmental stages (and less in defence), to increase success in canopy competition. Notably, *N. glauca* also has a thicker leaf epidermis covered by a prominent waxy cuticula with crystals [37,38], suggesting that it may invest in alternative defence strategies. Instead of relying on HGL-DTGs, *N. glauca* may employ a combination of physical defence and alternative chemical defence pathways that involve fatty alcohols delivered to the epicuticular wax layer [39].

The allopolyploid *N. benthamiana* is largely resistant to generalist herbivores due to the synthesis of acyl-sugars that are sequestered in trichomes [40]. In regard to the function of HGL-DTGs in this species, it will be interesting to investigate whether a specialised herbivore of *N. benthamiana* exists in the native distribution range of the species in Australia and whether it shows resistance to its alkaloids, similar to the reported interaction between *Manduca sexta* and *N. attenuata*. Discovery of such a (currently unknown) herbivore of *N. benthamiana* could substantiate the hypothesis that HGL-DTGs evolved as a defence against specialised herbivores.

The complexity of the HGL-DTG pathway and the large number of isomers lead us to assume that there are still undiscovered enzymes acting on HGL-DTGs. Previous studies reported that a rhamnose moiety can be linked by at least two glycosidic bonds to glucose, namely 4-1 and 6-1 [6,7]. Consequently, we hypothesise that two rhamnosyltransferases must exist for HGL-DTG synthesis. Heiling et al., 2021 [12] did not include three times rhamnosylated HGL-DTG in their study of virus-induced gene silencing of rhamnosyltransferase UGT91T1 from *N. attenuata* and *N. obtusifolia*. For the described di-rhamnosylated HGL-DTGs of *N. attenuata*, the rhamnosylations are terminal at different glucose moieties and connected by (4-1) glycosidic bonds [11,12]. As Heiling et al., 2021 [12] did not report (6-1) glycosidic bonds; it remains an open question whether or not there is a second rhamnosyltransferase type involved in the HGL-DTG pathway.

It also seems conceivable that two different glucosyltransferases are needed to glucosylate the two hydroxyl moieties of the 17HGL aglycone. One or more additional glucosyltransferase may exist that catalyse the formation of glucose-to-glucose attachments. The two potential glucosyltrasferases, UGT74P3 and UGT74P5, discovered in *N. attenuata* [12] would be consistent with the minimal requirement. Enzyme assays proved that NaUGT74P3, equivalent to UGT74P4 of *N. obtusifolia*, is able to attach a glucose moiety to both hydroxyls of 17HGL to form HGL-DTG 630.4, i.e., lyciumoside I [12]. NaUGT74P5 potentially mono-glucosylates the aglycone but does not tri-glucosylate HGL-DTGs in combination with UGT74P3 [12]. UGT74P5 could contribute to the observed variation of the isomer group 716.4 in our study by attaching glucose to a different position or possibly utilising a different hexose of identical mass (e.g., UDP galactose, which could serve as an alternative substrate). UGT74P5 may also act on structurally related specialised metabolites of *Nicotiana* species, such as triterpene glycosides, which are currently poorly investigated. Since UGT74P5 is not the glucosyltransferase forming glucosyl-glucose bonds, there could be an additional UGT present in *Nicotiana* species. Such an additional UGT remains to be discovered.

Regarding the functions of the different HGL-DTG moieties, HGL-DTG malonylations are lost upon pH change to pH 11 [10]. Such a pH change is typical of ingestion by insect larvae. Malonylations have been proposed as storage-related modifications that may have a protective function in planta [10]. The function of rhamnosylations is presently unknown. Glucose moieties have been shown to detoxify the HGL-DTGs in planta [12], whereas the deglycosylation in the insect activates the molecules’ toxicity [41]. At present, we neither understand why there is such a variety of differently glycosylated individual HGL-DTGs nor why there are rhamnosylations in addition to glucosylations, even though glucosylations are sufficient to avoid auto-toxicity within the plant [10]. It would be highly informative to subject the studied *Nicotiana* species to different species of herbivorous insects to study if and how the HGL-DTG profiles change qualitatively and/or quantitatively.

As an additional analytical aspect of our study on the HGL-DTG specialised metabolite family, we assessed the possibilities for annotation by fragmentation in ESI(-) ionisation mode mass spectrometry. Such mass spectra had not been described before for most HGL-DTGs. We have come to the conclusion that ESI(-) mode mass spectrometry is less informative for structure prediction than ESI(+) mode spectra, but the [M-H]^−^ ionisation product is reliably detectable in ESI(-) mode. We, therefore, believe that the ESI(-) ionisation mode is the better option to profile HGL-DTGs across experiments, not the least because the abundance of molecular ions is less diminished and variable by fragmentation reactions or abundant alternative adduct formation.

This study serves to generate hypotheses about the reaction paths that contribute to HGL-DTG biosynthesis. The proposed reaction scheme and reactions will require confirmation by enzymatic studies and future gene function investigations. To understand the structural complexity of HGL-DTGs, purification of isomers and NMR studies will become necessary to elucidate configurations of chemical bonds and to confirm the identity of the hexose and deoxyhexose moieties. Such detailed analyses will become feasible and motivated if future studies should reveal a specific accumulation of isomers or isomer sets in response to specific biotic or abiotic stresses or genetic modifications.

## 5. Conclusions

We characterised 17-hydroxygeranyllinalool diterpene glycoside (HGL-DTG) profiles of cultivated and wild *Nicotiana* species by detailed metabolome analysis using chromatographic retention and paired ESI(+) and ESI(-) mode mass spectrometry. We uncovered that the individual HGL-DTG profiles differ specifically between the investigated species but overlap extensively between the molecular model species *N. tabacum* and *N. benthamiana*. The shared ancestry of these species allows us to conclude the presence of similar central reaction schemes based on inferred chemical synthesis steps and structural relationships. In addition to the discovery of numerous isomeric conjugates, we revealed the HGL-DTG complexity of the *N. tabacum* cultivar Samsun NN and found it to be in stark contrast to the substantially lower complexity of this metabolite class in previously investigated *N. tabacum* cultivars. We propose that this striking case of metabolic diversity could be related to human selection in the breeding history of the cultivars. This information can potentially be used for more sustainable agriculture by selecting cultivars harbouring more insect deterrents. We point out that the mode of action of HGL-DTGs in plant defence, the diversity of the conjugated components, and the lack of information on key enzymatic steps warrant further studies. These will provide new insights into how and why the complex variety of HGL-DTGs is produced in different species of the genus, how the complexity evolved, how the pathway is regulated, and how it might be manipulated for agricultural purposes.

## Figures and Tables

**Table 1:** Annotated HGL-DTGs of *N. tabacum*, *N. glauca* and *N. benthamiana.* An internal order number (#) of this study is referenced in Table 2 and Table S3. The HGL-DTG name is composed of molecular mass and a lowercase letter indicating the elution sequence within a group of isomers. The putative molecular formula and decorations, as well as the previous nomenclature, are also given [11]. The boldface indicates newly described isomers. Previous nomenclature, according to [11], was extrapolated in these cases. The table is sorted by the putative number of hexoses (Hex), followed by the numbers of deoxyhexoses (DHex) and malonylations (Ma).

#	Name	Formula	Putative Decorations	Previous Nomenclature [11]
37	700.4 a	C_35_H_56_O_14_	1 Hex, 1 DHex, 1 Ma	DTG 718′
**59**	**700.4 b**	**C_35_H_56_O_14_**	**1 Hex, 1 DHex, 1 Ma**	**DTG 718** **″**
**64**	**700.4 c**	**C_35_H_56_O_14_**	**1 Hex, 1 DHex, 1 Ma**	**DTG 718‴**
43	630.4	C_32_H_54_O_12_	2 Hex	Lyciumoside I
24	776.4 a	C_38_H_64_O_16_	2 Hex, 1 DHex	DTG 794′
39	776.4 b	C_38_H_64_O_16_	2 Hex, 1 DHex	Lyciumoside IV
47	776.4 c	C_38_H_64_O_16_	2 Hex, 1 DHex	DTG 794‴
**29**	**862.4 a**	**C_41_H_66_O_19_**	**2 Hex, 1 DHex, 1Ma**	**DTG 880′**
38	862.4 b	C_41_H_66_O_19_	2 Hex, 1 DHex, 1Ma	DTG 880″
45	862.4 d	C_41_H_66_O_19_	2 Hex, 1 DHex, 1Ma	Nicotianoside Ib
46	862.4 c + d + e *	C_41_H_66_O_19_	2 Hex, 1 DHex, 1Ma	Nicotianoside Ia + b + c
50	862.4 e	C_41_H_66_O_19_	2 Hex, 1 DHex, 1Ma	Nicotianoside Ic
**36**	**948.4 a**	**C_44_H_68_O_22_**	**2 Hex, 1 Dhex, 2 Ma**	**DTG 966**
52	948.4 b	C_44_H_68_O_22_	2 Hex, 1 Dhex, 2 Ma	Nicotianoside IIb
58	948.4 c	C_44_H_68_O_22_	2 Hex, 1 Dhex, 2 Ma	Nicotianoside IIc
**3**	**716.4 a**	**C_35_H_56_O_15_**	**2 Hex, 1 Ma**	**DTG 734′**
**15**	**716.4 b**	**C_35_H_56_O_15_**	**2 Hex, 1 Ma**	**DTG 734″**
49	716.4 c	C_35_H_56_O_15_	2 Hex, 1 Ma	Nicotianoside IXc
54	716.4 d + e *	C_35_H_56_O_15_	2 Hex, 1 Ma	DTG 734‴ + DTG 734⁗
57	716.4 e	C_35_H_56_O_15_	2 Hex, 1 Ma	DTG 734⁗
60	716.4 e + f *	C_35_H_56_O_15_	2 Hex, 1 Ma	DTG 734⁗ + 734′′′′′
22	922.5 a	C_44_H_74_O_20_	2 Hex, 2 Dhex	DTG 940
31	922.5 b	C_44_H_74_O_20_	2 Hex, 2 Dhex	Nicotianoside III
28	1008.5 a	C_47_H_76_O_23_	2 Hex, 2 DHex, 1 Ma	DTG 1026′
35	1008.5 b	C_47_H_76_O_23_	2 Hex, 2 DHex, 1 Ma	DTG 1026″
42	1008.5 c	C_47_H_76_O_23_	2 Hex, 2 DHex, 1 Ma	Nicotianoside IV
**34**	**1094.5 a**	**C_50_H_78_O_26_**	**2 Hex, 2 DHex, 2 Ma**	**DTG 1112′**
**41**	**1094.5 b**	**C_50_H_78_O_26_**	**2 Hex, 2 DHex, 2 Ma**	**DTG 1112″**
**44**	**1094.5 c**	**C_50_H_78_O_26_**	**2 Hex, 2 DHex, 2 Ma**	**DTG 1112‴**
51	1094.5 d	C_50_H_78_O_26_	2 Hex, 2 Dhex, 2 Ma	Nicotianoside V
53	802.4 a	C_38_H_58_O_18_	2 Hex, 2 Ma	Nicotianoside Xb
55	802.4 a + b *	C_38_H_58_O_18_	2 Hex, 2 Ma	Nicotianoside Xb + c
62	802.4 c	C_38_H_58_O_18_	2 Hex, 2 Ma	Nicotianoside Xd
**63**	**802.4 d**	**C_38_H_58_O_18_**	**2 Hex, 2 Ma**	**Nicotianoside X**
21	1068.5	C_50_H_84_O_24_	2 Hex, 3 Dhex	DTG 1086
27	1154.5	C_53_H_86_O_27_	2 Hex, 3 DHex, 1 Ma	DTG 1172
6	938.5 a	C_44_H_74_O_21_	3 Hex, 1 Dhex	HGL-DTG 956
9	938.5 b	C_44_H_74_O_21_	3 Hex, 1 Dhex	Attenoside
7	1024.5 a	C_47_H_76_O_24_	3 Hex, 1 DHex, 1 Ma	DTG 1042
12	1024.5 b	C_47_H_76_O_24_	3 Hex, 1 Dhex, 1 Ma	HGL-DTG 1042
14	1024.5 c	C_47_H_76_O_24_	3 Hex, 1 Dhex, 1 Ma	Nicotianoside VIa
17	1024.5 d	C_47_H_76_O_24_	3 Hex, 1 Dhex, 1 Ma	Nicotianoside VIb
18	1024.5 e	C_47_H_76_O_24_	3 Hex, 1 Dhex, 1 Ma	Nicotianoside VIc
**20**	**1024.5 f**	**C_47_H_76_O_24_**	**3 Hex, 1 Dhex, 1 Ma**	**DTG 1042**
10	1110.5 a	C_50_H_78_O_27_	3 Hex, 1 DHex, 2 Ma	DTG 1128
23	1110.5 b	C_50_H_78_O_27_	3 Hex, 1 Dhex, 2 Ma	Nicotianoside VIIa
25	1110.5 c	C_50_H_78_O_27_	3 Hex, 1 Dhex, 2 Ma	Nicotianoside VIIb
30	1196.5 a	C_53_H_80_O_30_	3 Hex, 1 Dhex, 3 Ma	Nicotianoside VIIIa
33	1196.5 b	C_53_H_80_O_30_	3 Hex, 1 Dhex, 3 Ma	Nicotianoside VIIIb
**16**	**878.4 a**	**C_41_H_66_O_20_**	**3 Hex, 1 Ma**	**DTG 896′**
19	878.4 b + c *	C_41_H_66_O_20_	3 Hex, 1 Ma	Nicotianoside XIb+c
**40**	**878.4 d**	**C_41_H_66_O_20_**	**3 Hex, 1 Ma**	**DTG 896″**
1	1084.5 a	C_50_H_84_O_25_	3 Hex, 2 DHex	DTG 1102′
2	1084.5 b	C_50_H_84_O_25_	3 Hex, 2 DHex	DTG 1102″
**11**	**1084.5 c**	**C_50_H_84_O_25_**	**3 Hex, 2 DHex**	**DTG 1102‴**
4	1170.5 a	C_53_H_86_O_28_	3 Hex, 2 DHex, 1 Ma	DTG 1188′
5	1170.5 b	C_53_H_86_O_28_	3 Hex, 2 DHex, 1 Ma	DTG 1188″
8	1256.5 a	C_56_H_88_O_31_	3 Hex, 2 DHex, 2 Ma	DTG 1274′
13	1256.5 b	C_56_H_88_O_31_	3 Hex, 2 DHex, 2 Ma	DTG 1274″
26	964.4 a + b *	C_44_H_68_O_23_	3 Hex, 2 Ma	Nicotianoside XIIa + b
48	964.4 c	C_44_H_68_O_23_	3 Hex, 2 Ma	Nicotianoside XII
**56**	964.4 d + e *	**C_44_H_68_O_23_**	**3 Hex, 2 Ma**	**DTG 982″ + DTG 982‴**
**61**	**964.4 e**	**C_44_H_68_O_23_**	**3 Hex, 2 Ma**	**DTG 982‴**
32	1050.4	C_47_H_70_O_26_	3 Hex, 3 Ma	Nicotianoside XIIIa

* Isomers were not separated by automated data processing but manually distinguished.

**Table 2:** Comparisons to previous HGL-DTG annotations. HGL-DTGs are designated by internal order numbers (#) referencing to Table 1 and Table S3. Previously reported compound occurrence in Solanaceae species is indicated. The presence of HGL-DTGs in *N. attenuata* reference material is also indicated to support annotations through the classification of annotation levels. Identification levels according to [28] (1. IL: A–D) are: A by standard compound (not available here); B_(i)_ by MS-MS analysis; C by MS^n^ analyses (not available here); D by in-source MS analysis. HGL-DTG specific identification levels (2. IL: I–IV) are: IV aglycone mass, *m*/*z* 271.24 present within in-source spectrum; III *m*/*z* match with [11]; II *m*/*z* and approximate retention time match with [11]; I additional RT match to a *N. attenuata* reference sample. Potentially ambiguous isomer annotations are indicated. Ref.: Reference, IL—identification level, Glc—glucose, Rha—rhamnose, Ma—malonylation, n. d.—not described.

#	Species Name	Ref.	Structure Details [11]	Detected in *N. attenuata*	1. IL [28]	2. IL
37	*Nicotiana tomentosiformis*	[11]		no	D	II
59		n. d.		no	B_(i)_	II
64		n. d.		no	D	III
43	*Nicotiana attenuata*, *Lycium chinense*	[5]	Glc, Glc	yes	D	I
24	*Nicotiana alata*, *Capsicum* spp.	[11]		no	B_(i)_	II
39	*Nicotiana attenuata*, *N. africana*, *N. cavicola*, *N. obtusifolia*, *N. tomentosiformis*, *Lycium chinense*	[8]		yes	B_(i)_	I
47	*Nicotiana africana*, *N. tomentosiformis*	[11]		no	D	II
29		n. d.		no	B_(i)_	III
38	*Capsicum annuum*	[11]		no	B_(i)_	II
45	*Nicotiana attenuata*, *N. acuminata*, *N. africana*, *N. cavicola*, *N. clevelandii*, *N. obtusifolia*, *N. pauciflora*, *N. tomentosiformis*	[10]	Glc Ma (6-1), Glcc Rha (4-1)	yes	D	I
46	*Nicotiana attenuata*, *N. acuminata*, *N. africana*, *N. cavicola*, *N. clevelandii*, *N. obtusifolia*, *N. pauciflora*, *N. tomentosiformis*	[10]	Glc-Ma (6-1), Glc-Rha (4-1)	yes, ambigous	B_(i)_	II/I
50	*Nicotiana attenuata*	[10]	Glc-Ma (6-1), Glc-Rha (4-1)	yes	B_(i)_	I
36		n. d.		no	D	IV
52	*Nicotiana attenuata*, *N. acuminata*, *N. cavicola*, *N. clevelandii*, *N. obtusifolia*, *N. pauciflora*, *N. spegazzini*	[10]	Glc-Ma (6-1), Glc-Rha (4-1)-Ma (6-1)	yes	B_(i)_	I
58	*Nicotiana attenuata*, *N. acuminata*, *N. cavicola*, *N. clevelandii*, *N. obtusifolia*, *N. pauciflora*	[10]	Glc-Ma (6-1), Glc-Rha (4-1)-Ma (6-1)	yes, ambigous	B_(i)_	II/I
3		n. d.		no	D	III
15		n. d.		no	D	III
49	*Nicotiana attenuata*	[11]	Glc, Glc, Ma	yes	B_(i)_	I
54	*Lycium barbarum*	[11]		no	B_(i)_	II
57	*Lycium barbarum*	[11]		no	B_(i)_	II
60	*Lycium barbarum*	[11]		no	B_(i)_	II
22	*Nicotiana benthamiana*, *N. alata*, *Capsicum* spp.	[11]		no	B_(i)_	II
31	*Nicotiana attenuata*, *N. africana*, *N. cavicola*, *N. clevelandii*, *N. linearis*, *N. pauciflora*, *N. spegazzini*	[10]	Glc-Rha (4-1), Glc-Rha (4-1)	yes	D	I
28	*Nicotiana benthamiana*	[11]		no	B_(i)_	II
35	*Nicotiana benthamiana*, *Capsicum* spp.	[11]		no	B_(i)_	II
42	*Nicotiana attenuata/Nicotiana obtusifolia*, *N. acuminata*, *N. africana*, *N. cavicola*, *N. clevelandii*, *N. linearis*, *N. pauciflora*, *N. spegazzini*, *N. tomentosiformis*	[10]/[29]	Glc-Rha (4-1), Glc-Rha (4-1), Ma	yes	B_(i)_	I
34		n. d.		no	D	III
41		n. d.		no	D	III
44		n. d.		no	B_(i)_	III
51	*Nicotiana attenuata/Nicotiana obtusifolia*, *N. acuminata*, *N. africana*, *N. alata*, *N. cavicola*, *N. clevelandii*, *N. linearis*, *N. miersii*, *N. pauciflora*, *N. spegazzini*, *N. tomentosiformis*	[10]/[29]	Glc-Rha (4-1), Glc-Rha (4-1), 2 Ma	yes	B_(i)_	I
53	*Nicotiana attenuata*, *N. cavicola*, *N. obtusifolia*	[11]	Glc, Glc, 2 Ma	yes	B_(i)_	I
55	*Nicotiana attenuata*, *N. cavicola*, *N. obtusifolia*	[11]	Glc, Glc, 2 Ma	yes, ambigous	B_(i)_	II/I
62	*Nicotiana attenuata*	[11]	Glc, Glc, 2 Ma	yes	B_(i)_	I
63		n. d.		yes	D	III
21		[6]		no	B_(i)_	IV
27	*Nicotiana benthamiana*	[11]		no	B_(i)_	II
6	*Nicotiana attenuata*, *N. alata*, *N. cavicola*, *N. obtusifolia*	[11]	Glc-Glc (2-1), Glc-Rha (4-1)	yes	D	I
9	*Nicotiana attenuata*	[16]	Glc-Glc (2-1), Glc-Rha (4-1)	yes	D	I
7	*Capsicum annuum*	[11]		no	D	II
12	*Nicotiana attenuata*, *N. cavicola*, *Lycium barbarum*	[11]	Glc-Glc-Glc-Rha#, Ma	yes	D	I
14	*Nicotiana attenuata*, *N. acuminata*, *N. clevelandii*, *N. pauciflora*, *N. spegazzini*	[10]	Glc-Glc (2-1), Glc-Rha (4-1), 1 Ma	yes	D	I
17	*Nicotiana attenuata*, *N. acuminata*, *N. clevelandii*, *N. pauciflora*, *N. spegazzini*	[11]	Glc-Glc (2-1), Glc-Rha (4-1), 1 Ma	yes	D	I
18	*Nicotiana attenuata*, *N. clevelandii*	[11]	Glc-Glc (2-1), Glc-Rha (4-1), 1 Ma	yes	B_(i)_	I
20		n. d.		no	D	III
10	*Lycium barbarum*	[11]		no	B_(i)_	II
23	*Nicotiana attenuata*, *N. acuminata*, *N. clevelandii*, *N. pauciflora*, *N. spegazzini*	[10]	Glc-Glc (2-1), Glc-Rha (4-1), 2 Ma	yes	D	I
25	*Nicotiana attenuata*, *N. acuminata*, *N. cavicola*, *N. pauciflora*, *N. spegazzini*	[11]	Glc-Glc (2-1), Glc-Rha (4-1), 2 Ma	yes	B_(i)_	I
30	*Nicotiana attenuata*, *N. acuminata*, *N. clevelandii*, *N. pauciflora*, *N. spegazzini*	[11]	Glc-Glc (2-1), Glc-Rha (4-1), 3 Ma	yes	D	I
33	*Nicotiana attenuata*, *N. acuminata*	[11]	Glc-Glc (2-1), Glc-Rha (4-1), 3 Ma	yes	D	I
16		n. d.		no	B_(i)_	III
19	*Nicotiana attenuata*	[11]	Glc-Glc (2-1), Glc, Ma	yes	B_(i)_	I
40		n. d.		yes	B_(i)_	III
1	*Nicotiana alata*, *Capsicum* spp.	[11]		no	B_(i)_	II
2	*Capsicum* spp.	[11]		no	D	II
11		n. d.		no	D	III
4	*Nicotiana alata*, *Capsicum* spp.	[11]		no	D	II
5	*Nicotiana alata*, *Capsicum* spp.	[11]		no	B_(i)_	II
8	*Nicotiana alata*	[11]		no	D	II
13	*Nicotiana alata*	[11]		no	D	II
26	*Nicotiana attenuata*, *N. clevelandii*, *N. corymbosa*, *N. pauciflora*	[11]	Glc-Glc (2-1), Glc, 2 Ma	yes	B_(i)_	I
48	*Nicotiana acuminata*, *N. pauciflora*	[11]	Glc-Glc (2-1), Glc, 2 Ma	yes	D	II
56		n. d.		no	B_(i)_	III
61		n. d.		no	B_(i)_	III
32	*Nicotiana attenuata*, *N. quadrivalvis*	[11]	Glc-Glc (2-1), Glc, 3 Ma	yes	D	I

## Data Availability

Data utilized in the preparation of this manuscript are reported in the article and provided as Supplemental Materials (Supplemental Figures S1–S3, Tables S1–S4).

## Supplementary Materials

The following supporting information can be downloaded at: https://www.mdpi.com/article/10.3390/metabo14100562/s1; Figure S1: Exemplary images of the studied plants; Figure S2: 17HGL aglycone exemplary positive mode MS/MS spectra; Figure S3: Fragmentation of HGL-DTG 862.4 e (nicotianoside Ic); Table S1: Fragment interpretation of HGL-DTG 862.4 e (nicotianoside Ic); Table S2: Fragment interpretation of HGL-DTG 1170.5 b (DTG 1188′′); Table S3: RT average, range and *m*/*z* errors of annotated HGL-DTGs; Table S4: Representative MS/MS spectra information of each HGL-DTG isomer group.
