# Supplementary material for: Detailed Profiling of 17-Hydroxygeranyllinalool Diterpene Glycosides from Nicotiana Species Reveals Complex Reaction Networks of Conjugation Isomers
Source: Metabolites. 2024 Oct 20;14(10):562. doi: 10.3390/metabo14100562 (PMC11509208; doi:10.3390/metabo14100562)
Supplement: Supplementary file 1 [file metabolites-14-00562-s001.zip › metabolites-3219201-supplementary.pdf]

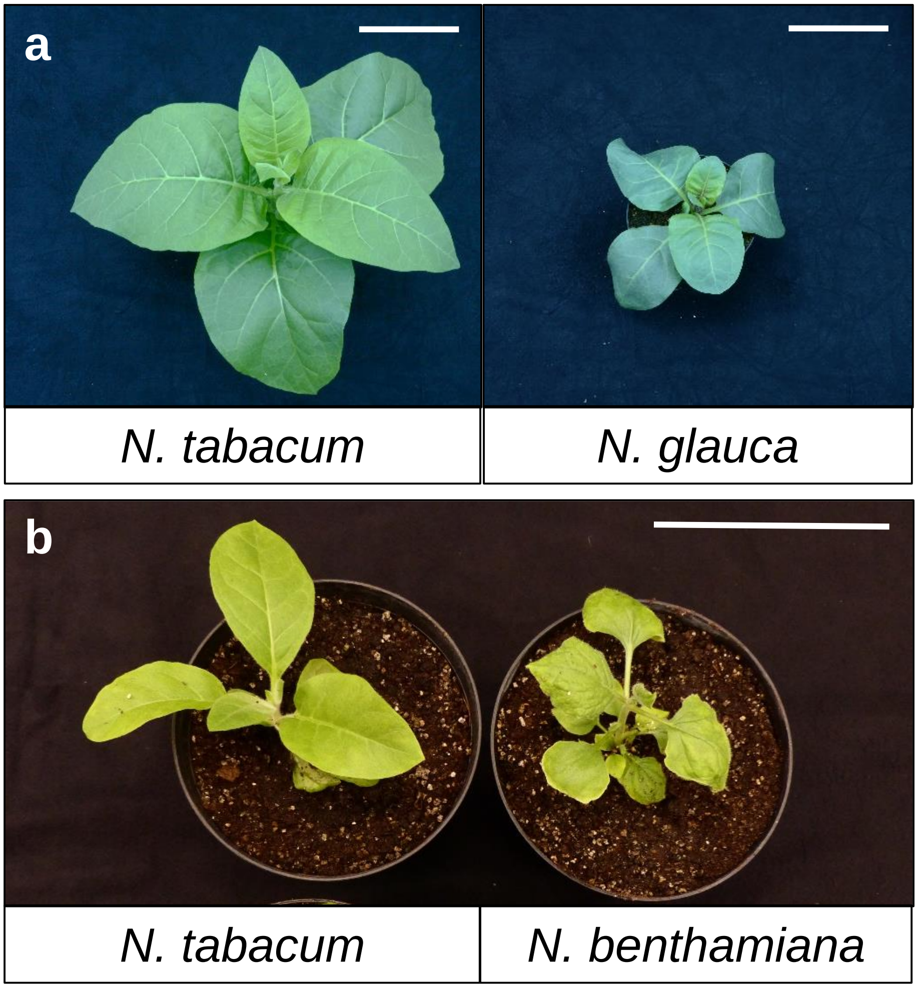

Figure S1. Exemplary images of plant species used and their appearance in the two different experiments, where plants originated from seeds (**a**) and sterile culture tissue (**b**). The white scale bar in the upper right-hand corner represents 10 cm. Pictures were taken in independent sessions with different illumination conditions at a developmental stage when the plants had 7–10 fully expanded leaves.

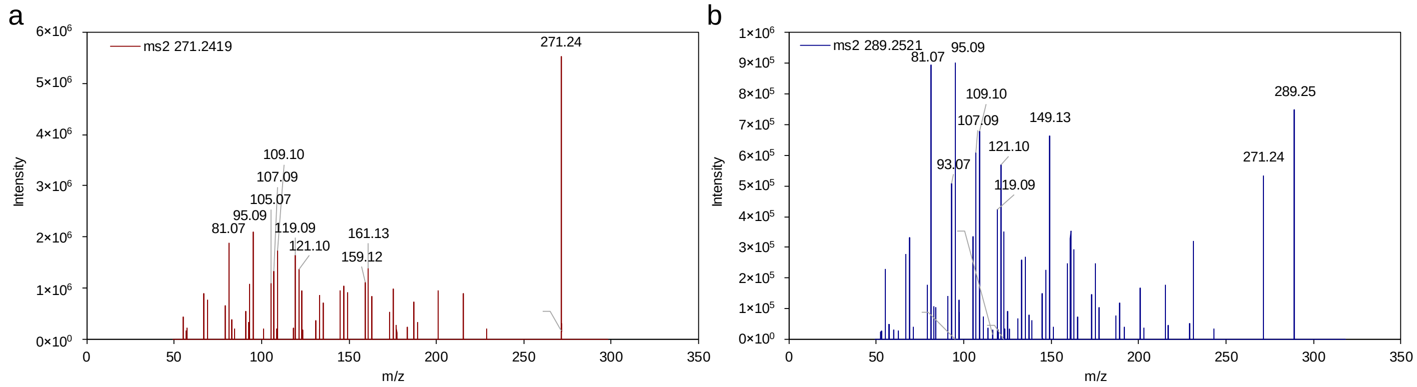

Figure S2. Exemplary MS/MS spectra of the 17HGL aglycone without hydroxyl groups ($C_{20}H_{31}$+, m/z 271.2420, red, **a**) and with one remaining hydroxyl group ($C_{20}H_{33}O$+, m/z 289.2526, blue, **b**) from positive ionisation mode mass spectrometry. Spectra are taken from a *N. tabacum* cv. Samsun NN shoot sample. Spectra are extracted at scans from retention time 12.11 min (a) and 12.09 min (b) with parent m/z reported by the insert (left top). The top ten most abundant MS/MS fragments are indicated by mass to charge ratios (m/z). Reported intensities have arbitrary units.

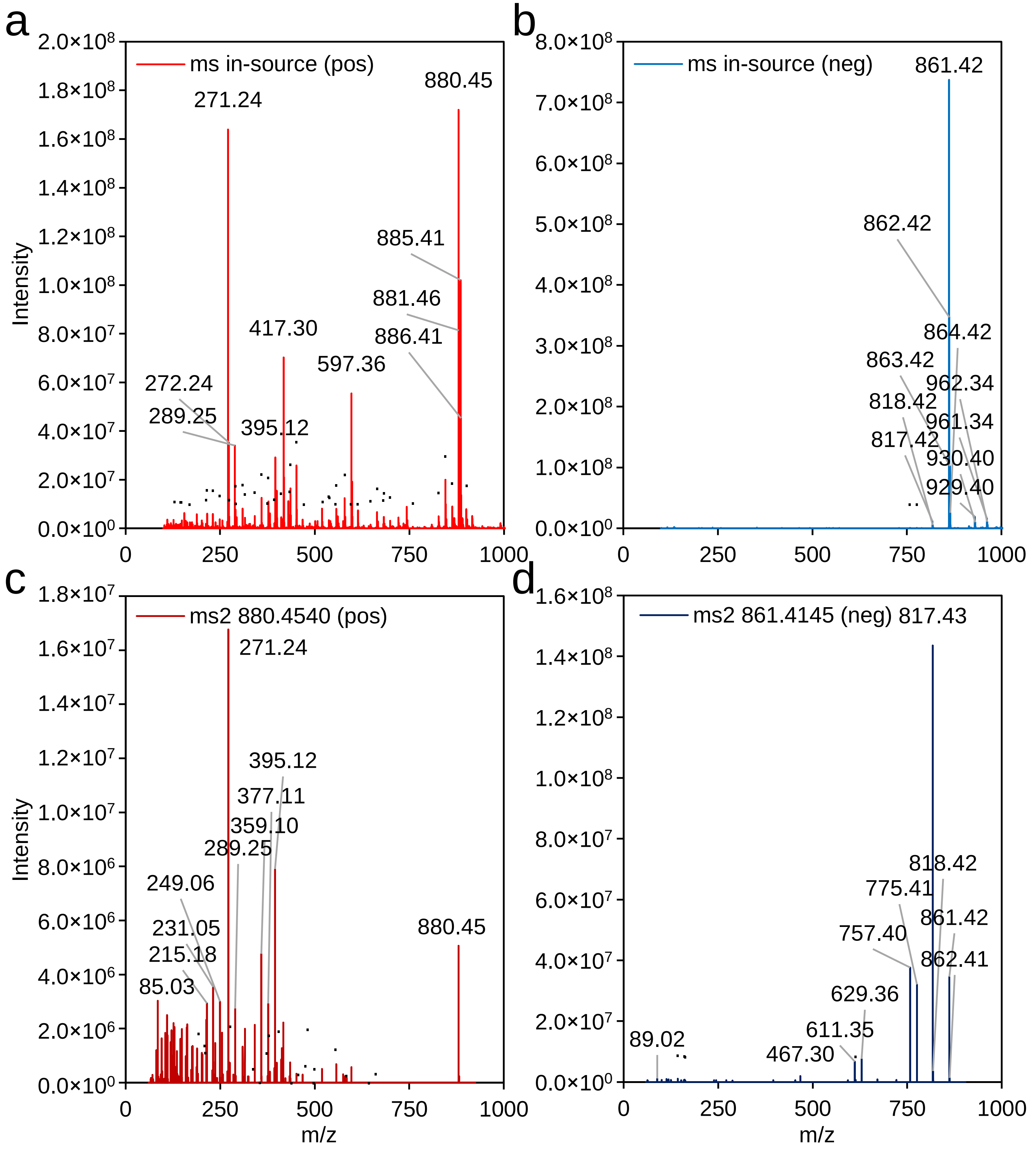

Figure S3. Exemplary fragmentation analysis of HGL-DTG 862.4 e, i.e. nicotianoside Ic (Table 1), by positive mode ionisation (**a**, **c**, red) and negative mode ionisation (**b**, **d**, blue) mass spectrometry. Fragment ions of in-source fragments (**a**, **b**) and from MS/MS (ms2) fragmentation experiments at collision energy = 25 eV (**c**, **d**) are compared. Data are from the same sample of *N. tabacum* cv. Samsun NN. Mass spectra were extracted at retention time 12.09 min (ESI(+) mode) and 12.08 min (ESI(-) mode), respectively. The top ten most abundant fragments are indicated by mass to charge ratios (m/z). Further annotated fragments (Table S1) are labelled by dots. Reported intensities have arbitrary units.

Table S1. Analysis of HGL-DTG 862.4 e (nicotianoside Ic) with annotations of observed mass fragments (Figure S2). MS/ MS mass spectra were recorded using the parent ion m/z 880.4540 in positive and m/z 861.4145 in negative mode at collision energy 25 eV. Molecular formula, predicted theoretical m/z, and measured m/z with m/z errors are listed. Isotopologues are indicated by prefixes indicating the nominal mass shift. Ion. mode: Ionisation mode; Aglyc: Aglycone ($C_{20}H_{34}O_2$); Rha: Rhamnose ($C_6H_{12}O_5$) ; Glc: Glucose ($C_6H_{12}O_6$); Ma: Malonic acid ($C_3H_4O_4$).

Ion. mode	group	fragment	molecular formula	theoretical *m/z*	in-source *m/z*	*m/z* error (ppm)	MS/MS *m/z*	*m/z* error (ppm)
	agly-cone	[Aglyc-2H_2O+H]$^+$	$C_{20}H_{31}$+	271.2420	271.2421	0.05	271.2404	-1.62
		[Aglyc-H_2O+H]$^+$	$C_{20}H_{33}O$+	289.2526	289.2524	-0.15	289.2541	1.52
	sugar fragments	[Rha-H2O+H]+	$C_6H_{11}O_4$+	147.0652	147.0649	-0.32	147.0649	-0.31
		[Rha-2H2O+H]+	$C_6H_9O_3$+	129.0546	129.0545	-0.09	129.0551	0.43
		[Glc-2H2O+H]+	$C_6H_9O_4$+	145.0495	145.0495	-0.05	145.0493	-0.21
		[Glc+Ma-2H2O+H]+	$C_9H_{13}O_8$+	249.0605	249.0606	0.08	249.0594	-1.11
		[Glc+Ma-3H2O+H]+	$C_9H_{11}O_7$+	231.0499	231.0497	-0.24	231.0491	-0.84
		[Glc+Ma-4H2O+H]+	$C_9H_9O_6$+	213.0394	213.0388	-0.57	213.0390	-0.36
		[Glc+Ma-4H2O-CO2+H]+	$C_8H_9O_4$+	169.0495	169.0495	-0.04	NA	NA
		[Rha+Glc-2H2O+H]+	$C_{12}H_{21}O_9$+	309.1180	309.1181	0.05	309.1195	1.46
		[Rha+Glc-3H2O+H]+	$C_{12}H_{19}O_8$+	291.1074	291.1066	-0.82	291.1072	-0.28
		[Rha+Glc-4H2O+H]+	$C_{12}H_{17}O_7$+	273.0969	273.0966	-0.27	273.0984	1.52
		[Rha+Glc+Ma-3H2O+H]+	$C_{15}H_{23}O_{12}$+	395.1184	395.1189	0.47	395.1220	3.59
		[Rha+Glc+Ma-4H2O+H]+	$C_{15}H_{21}O_{11}$+	377.1078	377.1083	0.47	377.1091	1.25
		[Rha+Glc+Ma-5H2O+H]+	$C_{15}H_{19}O_{10}$+	359.0973	359.0967	-0.62	359.0976	0.29
		[Rha+Glc+Ma-5H2O-CO2+H]+	$C_{14}H_{19}O_8$+	315.1074	315.1070	-0.45	315.1066	-0.81
		[Rha+Glc+Ma-6H2O+H]+	$C_{15}H_{17}O_9$+	341.0867	341.0858	-0.92	341.0884	1.71
		[Glc+Glc+Ma-3H2O+H]+	$C_{15}H_{23}O_{13}$+	411.1133	411.1124	-0.94	411.1134	0.13
		[Glc+Glc+Ma-4H2O+H]+	$C_{15}H_{21}O_{12}$+	393.1028	393.1023	-0.47	393.1049	2.13
		[Glc+Glc+Ma-5H2O+H]+	$C_{15}H_{19}O_{11}$+	375.0922	375.0919	-0.28	375.0909	-1.29
		[Rha+Glc+Glc-3H2O+H]+	$C_{18}H_{31}O_{14}$+	471.1708	471.1713	0.44	NA	
		[Rha+Glc+Glc+Ma-4H2O+H]+	$C_{21}H_{33}O_{17}$+	557.1712	557.1715	0.25	557.1695	-1.75
		[Rha+Glc+Glc+Ma-5H2O+H]+	$C_{21}H_{31}O_{16}$+	539.1607	539.1594	-1.25	NA	NA
		[Rha+Glc+Glc+Ma-6H2O+H]+	$C_{21}H_{29}O_{15}$+	521.1501	521.1511	1.01	NA	NA
	sugar + malonyl losses	[Aglyc+Rha-3H_2O+H]$^+$	$C_{26}H_{41}O_4$+	417.2999	417.3005	0.58	417.3038	3.86
		[Aglyc+Rha-2H_2O+H]$^+$	$C_{26}H_{43}O_5$+	435.3105	435.3110	0.46	435.3155	5.02
		[Aglyc+Glc-3H_2O+H]$^+$	$C_{26}H_{41}O_5$+	433.2949	433.2936	-1.24	433.2988	3.97
		[Aglyc+Glc-2H_2O+H]$^+$	$C_{26}H_{43}O_6$+	451.3054	451.3063	0.91	451.3096	4.15
		[Aglyc+Glc+Ma-3H_2O+H]$^+$	$C_{29}H_{45}O_9$+	537.3058	537.3062	0.41	NA	NA
		[Aglyc+Glc+Ma-2H_2O+H]$^+$	$C_{29}H_{47}O_{10}$+	555.3164	555.3145	-1.85	NA	NA
		[Aglyc+Rha+Glc-4H_2O+H]$^+$	$C_{32}H_{51}O_9$+	579.3528	579.3512	-1.56	579.3656	12.81
		[Aglyc+Rha+Glc-3H_2O+H]$^+$	$C_{32}H_{53}O_{10}$+	597.3633	597.3643	0.93	597.3653	1.97
		[Aglyc+Rha+Glc+Ma-6H_2O+H]$^+$	$C_{35}H_{51}O_{11}$+	647.3426	647.3428	0.20	NA	NA
positive		[Aglyc+Rha+Glc+Ma-5H_2O+H]$^+$	$C_{35}H_{53}O_{12}$+	665.3532	665.3542	1.00	NA	NA

		$[Aglyc+Rha+Glc+Ma-4H_2O+H]^+$	$C_{35}H_{55}O_{13}+$	683.3637	683.3644	0.66	NA	NA
		$[Aglyc+Glc+Glc-4H_2O+H]^+$	$C_{32}H_{51}O_{10}+$	595.3477	595.3476	-0.09	NA	NA
		$[Aglyc+Glc+Glc-3H_2O+H]^+$	$C_{32}H_{53}O_{11}+$	613.3582	613.3569	-1.34	NA	NA
		$[Aglyc+Glc+Glc+Ma-5H_2O+H]^+$	$C_{35}H_{53}O_{13}+$	681.3481	681.3479	-0.21	NA	NA
		$[Aglyc+Glc+Glc+Ma-4H_2O+H]^+$	$C_{35}H_{55}O_{14}+$	699.3586	699.3586	-0.01	NA	NA
		$[Aglyc+Rha+Glc+Glc-4H_2O+H]^+$	$C_{38}H_{63}O_{15}+$	759.4161	759.4164	0.27	NA	NA
		$[Aglyc+Rha+Glc+Glc+Ma-6H_2O+H]^+$	$C_{41}H_{63}O_{17}+$	827.4060	827.4043	-1.64	NA	NA
		$[Aglyc+Rha+Glc+Glc+Ma-5H_2O+H]^+$	$C_{41}H_{65}O_{18}+$	845.4165	845.4185	1.95	NA	NA
	**adducts**	$[M+H]^+$	$C_{41}H_{67}O_{19}+$	863.4271	863.4251	-1.96	NA	NA
		$[M+NH_4]^+$	$C_{41}H_{70}O_{19}N+$	880.4537	880.4517	-1.94	880.4466	-7.02
		$[M+NH_4]^+$ isotopologue	$^{1+}[C_{41}H_{70}O_{19}N+]$	881.4623	881.4557	-6.59	881.4383	-23.98
		$[M+Na]^+$	$C_{41}H_{66}O_{19}Na+$	885.4091	885.4093	0.27	NA	NA
		$[M+Na]^+$ isotopologue	$^{1+}[C_{41}H_{66}O_{19}Na+]$	886.4177	886.4095	-8.24	NA	NA
		$[M+K]^+$	$C_{41}H_{66}O_{19}K+$	901.3830	901.3829	-0.11	NA	NA
**negative**	**losses**	$[M-H]^-$	$C_{41}H_{65}O_{19}-$	861.4126	861.4159	3.32	861.4236	11.03
		$[M-H]^-$ isotopologue	$^{1+}[C_{41}H_{65}O_{19}-]$	862.4212	862.4182	-2.97	862.4079	-13.36
		$[M-H]^-$ isotopologue	$^{2+}[C_{41}H_{65}O_{19}-]$	863.4299	863.4214	-8.53	NA	NA
		$[M-H]^-$ isotopologue	$^{3+}[C_{41}H_{65}O_{19}-]$	864.4385	864.4205	-18.00	NA	NA
		$[M-CO_2-H]^-$	$C_{40}H_{65}O_{17}-$	817.4227	817.4217	-0.99	817.4306	7.84
		$[M-CO_2-H]^-$ isotopologue	$^{1+}[C_{40}H_{65}O_{17}-]$	818.4314	818.4249	-6.50	818.4204	-10.97
	**fragments**	$[Ma-O+H]^-$	$C_3H_3O_4-$	89.0244	NA	NA	89.0221	-2.30
		$[Rha-H]^-$	$C_6H_{11}O_5-$	163.0612	NA	NA	163.0580	-3.17
		$[Glc-H_2O-H]^-$	$C_6H_9O_5-$	161.0455	NA	NA	161.0428	-2.72
		$[Glc-2H_2O-H]^-$	$C_6H_7O_4-$	143.0350	NA	NA	143.0334	-1.62
	**sugar + malonyl losses**	$[Aglyc+Glc+Glc+Rha-4H_2O-H]^-$	$C_{38}H_{62}O_{15}-$	757.4016	757.4056	4.01	757.4035	1.92
		$[Aglyc+Glc+Glc+Rha-3H_2O-H]^-$	$C_{38}H_{63}O_{16}-$	775.4122	775.4149	2.70	775.4114	-0.80
		$[Aglyc+Glc+Glc-3H_2O-H]^-$	$C_{32}H_{51}O_{11}-$	611.3437	NA	NA	611.3496	5.88
		$[Aglyc+Glc+Glc-2H_2O-H]^-$	$C_{32}H_{53}O_{12}-$	629.3543	NA	NA	629.3567	2.41
		$[Aglyc+Glc+Rha-2H_2O-H]^-$	$C_{32}H_{53}O_{11}-$	613.3593	NA	NA	613.3586	-0.76
		$[Aglyc+Glc-H_2O-H]^-$	$C_{26}H_{43}O_7-$	467.3014	NA	NA	467.2995	-1.98

Table S2. Analysis of HGL-DTG 1170.5 b (DTG 1188'') with annotations of observed mass fragments (Figure 3). MS/ MS mass spectra were recorded using the parent ion m/z 1188.5641 in positive and m/z 1169.5238 in negative mode at collision energy 25 eV. Molecular formula, predicted theoretical m/z, and measured m/z with m/z errors are listed. Isotopologues are indicated by prefixes indicating the nominal mass shift. Ion. mode: Ionisation mode; Aglyc: Aglycone ($C_{20}H_{34}O_2$); DHex: Deoxyhexose ($C_6H_{12}O_5$); Hex: Hexose ($C_6H_{12}O_6$); Ma: Malonic acid ($C_3H_4O_4$).

Ion. mode	group	fragment	sum formula	theoretical *m/z*	in-source *m/z*	*m/z* error (ppm)	MS/MS *m/z*	*m/z* error (ppm)
positive	aglycone	[Aglyc-2H_2O+H]$^+$	$C_{20}H_{31}$+	271.2420	271.2419	-0.18	271.2441	2.03
		[Aglyc-H_2O+H]$^+$	$C_{20}H_{33}O$+	289.2526	289.2521	-0.46	289.2555	2.89
	sugar fragments	[DHex-H_2O+H]$^+$	$C_6H_{11}O_4$+	147.0652	147.0651	-0.06	147.0654	0.26
		[DHex-2H_2O+H]$^+$	$C_6H_9O_3$+	129.0546	129.0524	-2.25	129.0554	0.82
		[Hex-2H_2O+H]$^+$	$C_6H_9O_4$+	145.0495	145.0494	-0.09	NA	NA
		[Hex+Ma-2H_2O+H]$^+$	$C_9H_{13}O_8$+	249.0605	249.0607	0.17	NA	NA
		[Hex+Ma-3H_2O+H]$^+$	$C_9H_{11}O_7$+	231.0499	231.0505	0.61	NA	NA
		[Hex+Ma-4H_2O-CO_2+H]$^+$	$C_8H_9O_4$+	169.0495	169.0495	0.00	NA	NA
		[DHex+DHex-2H_2O+H]$^+$	$C_{12}H_{21}O_8$+	293.1231	293.1225	-0.59	293.1228	-0.33
		[DHex+DHex-3H_2O+H]$^+$	$C_{12}H_{19}O_7$+	275.1125	275.1110	-1.55	NA	NA
		[DHex+DHex-4H_2O+H]$^+$	$C_{12}H_{17}O_6$+	257.1020	257.1012	-0.74	257.1047	2.77
		[DHex+Hex-2H_2O+H]$^+$	$C_{12}H_{21}O_9$+	309.1180	309.1179	-0.09	309.1181	0.10
		[DHex+Hex-4H_2O+H]$^+$	$C_{12}H_{17}O_7$+	273.0969	273.0967	-0.19	273.0964	-0.44
		[DHex+Hex+Ma-3H_2O+H]$^+$	$C_{15}H_{23}O_{12}$+	395.1184	395.1180	-0.36	395.1162	-2.17
		[DHex+Hex+Ma-4H_2O+H]$^+$	$C_{15}H_{21}O_{11}$+	377.1078	377.1085	0.67	377.1111	3.24
		[DHex+Hex+Ma-5H_2O+H]$^+$	$C_{15}H_{19}O_{10}$+	359.0973	359.0968	-0.45	359.0992	1.96
		[DHex+Hex+Ma-5H_2O-CO_2+H]$^+$	$C_{14}H_{19}O_8$+	315.1074	315.1084	1.00	NA	NA
		[DHex+Hex+Ma-6H_2O+H]$^+$	$C_{15}H_{17}O_9$+	341.0867	341.0874	0.71	NA	NA
		[Hex+Hex-4H_2O+H]$^+$	$C_{12}H_{17}O_8$+	289.0918	289.0904	-1.35	NA	NA
		[Hex+Hex+Ma-3H_2O+H]$^+$	$C_{15}H_{23}O_{13}$+	411.1133	411.1145	1.21	411.1174	4.10
		[DHex+DHex+Hex-3H_2O+H]$^+$	$C_{18}H_{31}O_{13}$+	455.1759	455.1777	1.77	455.1810	5.08
		[DHex+DHex+Hex-5H_2O+H]$^+$	$C_{18}H_{27}O_{11}$+	419.1548	419.1543	-0.54	NA	NA
		[DHex+DHex+Hex+Ma-4H_2O+H]$^+$	$C_{21}H_{33}O_{16}$+	541.1763	NA	NA	541.1821	5.76
		[DHex+Hex+Hex-3H_2O+H]$^+$	$C_{18}H_{31}O_{14}$+	471.1708	471.1706	-0.20	471.1675	-3.29
		[DHex+Hex+Hex-5H_2O+H]$^+$	$C_{18}H_{27}O_{12}$+	435.1497	435.1496	-0.078	435.1527	3.04
		[DHex+Hex+Hex+Ma-4H_2O+H]$^+$	$C_{21}H_{33}O_{17}$+	557.1712	557.1717	0.46	557.1823	11.05
		[DHex+Hex+Hex+Ma-5H_2O+H]$^+$	$C_{21}H_{31}O_{16}$+	539.1607	539.1626	1.96	NA	NA
		[DHex+DHex+DHex+Hex+Ma-7H_2O-CO_2+H]$^+$	$C_{26}H_{39}O_{16}$+	607.2233	607.2193	-3.98	NA	NA
		[DHex+DHex+Hex+Hex-4H_2O+H]$^+$	$C_{24}H_{41}O_{18}$+	617.2287	617.2288	0.10	617.2342	5.46
		[DHex+DHex+Hex+Hex-5H_2O+H]$^+$	$C_{24}H_{39}O_{17}$+	599.2182	599.2138	-4.42	NA	NA
	sugar + malonyl losses	[Aglyc+DHex-3H_2O+H]$^+$	$C_{26}H_{41}O_4$+	417.2999	417.3004	0.43	417.3044	4.42
		[Aglyc+DHex-2H_2O+H]$^+$	$C_{26}H_{43}O_5$+	435.3105	435.3107	0.22	435.3164	5.87
		[Aglyc+Hex-3H_2O+H]$^+$	$C_{26}H_{41}O_5$+	433.2949	433.2938	-1.02	NA	NA
		[Aglyc+Hex-2H_2O+H]$^+$	$C_{26}H_{43}O_6$+	451.3054	451.3068	1.35	NA	NA

		[Aglyc+Hex+Ma-3H_2O+H]$^+$	$C_{29}H_{43}O_{8}+$	537.3058	537.3003	-5.50	NA	NA
		[Aglyc+Hex+Ma-2H_2O+H]$^+$	$C_{29}H_{45}O_{9}+$	555.3164	555.3176	1.19	NA	NA
		[Aglyc+DHex+DHex-4H_2O+H]$^+$	$C_{32}H_{51}O_{8}+$	563.3578	563.3588	0.96	NA	NA
		[Aglyc+DHex+DHex-3H_2O+H]$^+$	$C_{32}H_{53}O_{9}+$	581.3684	581.3669	-1.46	NA	NA
		[Aglyc+DHex+Hex-4H_2O+H]$^+$	$C_{32}H_{51}O_{9}+$	579.3528	579.3547	1.94	NA	NA
		[Aglyc+DHex+Hex-3H_2O+H]$^+$	$C_{32}H_{53}O_{10}+$	597.3633	597.3651	1.73	NA	NA
		[Aglyc+Hex+Hex-4H_2O+H]$^+$	$C_{32}H_{51}O_{10}+$	595.3477	595.3477	0.05	NA	NA
		[Aglyc+Hex+Hex-3H_2O+H]$^+$	$C_{32}H_{53}O_{11}+$	613.3582	613.3570	-1.19	NA	NA
		[Aglyc+Hex+Hex+Ma-5H_2O+H]$^+$	$C_{35}H_{53}O_{13}+$	681.3481	681.3487	0.64	NA	NA
		[Aglyc+DHex+DHex+Hex-5H_2O+H]$^+$	$C_{38}H_{61}O_{13}+$	725.4107	725.4072	-3.52	NA	NA
		[Aglyc+DHex+DHex+Hex-4H_2O+H]$^+$	$C_{38}H_{63}O_{14}+$	743.4212	743.4224	1.18	743.4151	-6.17
		[Aglyc+DHex+Hex+Hex-5H_2O+H]$^+$	$C_{38}H_{61}O_{14}+$	741.4056	741.4085	2.94	NA	NA
		[Aglyc+DHex+Hex+Hex-4H_2O+H]$^+$	$C_{38}H_{63}O_{15}+$	759.4161	759.4168	0.64	NA	NA
		[Aglyc+DHex+DHex+Hex+Hex-6H_2O+H]$^+$	$C_{44}H_{71}O_{18}+$	887.4635	887.4615	-1.96	NA	NA
		[Aglyc+DHex+DHex+Hex+Hex-5H_2O+H]$^+$	$C_{44}H_{73}O_{19}+$	905.4741	905.4714	-2.66	905.4733	-0.74
		[Aglyc+DHex+DHex+Hex+Hex+Hex+Ma-7H_2O+H]$^+$	$C_{53}H_{85}O_{27}+$	1153.5273	1153.5302	2.94	NA	NA
	adducts	[M+H]$^+$	$C_{53}H_{87}O_{28}+$	1171.5378	1171.5368	-1.02	NA	NA
		[M+NH4]$^+$	$C_{53}H_{90}O_{28}N+$	1188.5644	1188.5689	4.56	1188.5522	-12.19
		[M+NH4]$^+$ isotopologue	$^{1+}[C_{53}H_{90}O_{28}N+]$	1189.5731	1189.5702	-2.82	1189.5627	-10.37
		[M+Na]$^+$	$C_{53}H_{86}O_{28}Na+$	1193.5198	1193.5209	1.12	NA	NA
		[M+K]$^+$	$C_{53}H_{86}O_{28}K+$	1209.4937	1209.4897	-4.03	NA	NA
negative	losses	[M-H]$^-$	$C_{53}H_{85}O_{28}-$	1169.5233	1169.5279	4.63	1169.5378	14.53
		[M-H]$^-$ isotopologue	$^{1+}[C_{53}H_{85}O_{28}-]$	1170.5320	1170.5247	-7.27	1170.5155	-16.44
		[M-CO_2-H]$^-$	$C_{52}H_{85}O_{26}-$	1125.5335	NA	NA	1125.5275	-6.00
		[M-CO_2-H]$^-$ isotopologue	$^{1+}[C_{52}H_{85}O_{26}-]$	1126.5421	NA	NA	1126.5156	-26.53
	sugar fragments	[DHex-H_2O-H]$^-$	$C_{6}H_{9}O_{4}-$	145.0506	145.0495	-1.13	NA	NA
		[Hex-H]$^-$	$C_{6}H_{11}O_{6}-$	179.0561	179.0553	-0.86	NA	NA
		[Hex-H_2O-H]$^-$	$C_{6}H_{9}O_{5}-$	161.0455	161.0445	-1.03	NA	NA
		[Hex-2H_2O-H]$^-$	$C_{6}H_{7}O_{4}-$	143.0350	143.0340	-1.03	NA	NA
	sugar + malonyl losses	[Aglyc+Hex+Hex+Hex+DHex+DHex-6H_2O-H]$^-$	$C_{50}H_{81}O_{24}-$	1065.5123	NA	NA	1065.5132	0.87
		[Aglyc+Hex+Hex+Hex+DHex+DHex-5H_2O-H]$^-$	$C_{50}H_{83}O_{25}-$	1083.5229	NA	NA	1083.5097	-13.17
		[Aglyc+Hex+Hex+DHex+Ma-4H_2O-H]$^-$	$C_{41}H_{65}O_{19}-$	861.4126	861.4105	-2.01	NA	NA
		[Aglyc+Hex+Hex+Hex+Ma-4H_2O-H]$^-$	$C_{41}H_{65}O_{20}-$	877.4075	877.4082	0.77	NA	NA
		[Aglyc+Hex+Hex+Ma-4H_2O-H]$^-$	$C_{35}H_{53}O_{14}-$	713.3390	713.3386	-0.41	NA	NA
		[Aglyc+Hex+Hex+Ma-3H_2O-H]$^-$	$C_{35}H_{55}O_{15}-$	715.3546	715.3530	-1.68	NA	NA

Table S3. Experimental information of annotated HGL-DTGs. The average retention time and retention time range of chromatograms recorded in negative, ESI(-), and positive ionisation mode, ESI(+) from two experiments are reported. The measured and expected m/z are listed with measurement accuracy (error). Available MS/MS information of $[M-H]^-$ and $[M+NH_4]^+$ (Table S4) is indicated. Failing automated retention time assignments and required manual curation of elution sequences is indicated by "0.00 0-0". # – internal order number, RT – retention time, av. – average, Exp1 – experiment 1, Exp2 – experiment 2, theor. – theoretical, spec. – spectrum, avail. – available, n. d. – not detected.

general					$[M+NH_4]^+$				$[M-H]^-$			
#	RT av. Exp1	RT range Exp1	RT av. Exp2	RT range Exp2	ESI(+) theor. m/z	ESI(+) detected m/z	m/z error (ppm)	MS^2 spec. avail.	ESI(-) theor. m/z	ESI(-) detected m/z	m/z error (ppm)	MS^2 spec. avail.
37	11.78	11.73-11.85	11.78	11.72-11.86	718.401	718.403	1.8		699.360	699.361	1.0	
59	n. d.	n. d.	12.24	12.18-12.29	718.401	718.400	-1.1		699.360	699.360	0.6	
64	12.93	12.89-13.03	12.93	12.89-13.01	718.401	718.401	0.2		699.360	699.363	3.4	yes
43	11.89	11.83-11.97	11.89	11.84-11.98	648.395	648.397	1.6		629.354	629.353	-0.9	yes
24	11.34	11.25-11.45	11.35	11.25-11.48	794.453	794.454	0.7	yes	775.412	775.413	1.2	
39	11.80	11.73-11.9	11.80	11.73-11.87	794.453	794.455	2.1	yes	775.412	775.413	0.7	yes
47	n. d.	n. d.	12.03	11.94-12.13	794.453	794.453	-0.1		775.412	775.410	-1.7	
29	0.00	0-0	11.60	11.49-11.68	880.454	880.455	1.2	yes	861.413	861.413	0.1	
38	n. d.	n. d.	11.79	11.74-11.84	880.454	880.452	-1.7	yes	861.413	861.414	1.2	
45	0.00	0-0	0.00	0-0	880.454	0.000			861.413	0.000		
46	12.03	11.93-12.12	12.03	11.89-12.12	880.454	880.454	0.5	yes	861.413	861.414	1.3	yes
50	0.00	0-0	0.00	0-0	880.454	0.000		yes	861.413	0.000		
36	11.77	11.69-11.85	n. d.	n. d.	966.454	966.456	1.7		947.413	947.415	1.6	
52	12.14	12.09-12.21	12.15	12.08-12.2	966.454	966.456	1.6	yes	947.413	947.412	-1.1	yes
58	12.23	12.2-12.28	12.24	12.19-12.29	966.454	0.000		yes	947.413	947.412	-1.1	
3	10.62	10.58-10.67	10.62	10.5-10.68	734.396	734.399	2.9		715.355	715.355	-0.1	
15	n. d.	n. d.	11.11	11.07-11.15	734.396	734.395	-0.3		715.355	715.353	-1.2	
49	12.07	12-12.14	12.07	12.02-12.15	734.396	734.398	2.0	yes	715.355	715.356	1.8	yes
54	0.00	0-0	12.15	12.15-12.27	734.396	734.395	-0.4	yes	715.355	0.000		
57	0.00	0-0	0.00	0-0	734.396	0.000			715.355	0.000		
60	12.28	12.24-12.38	12.27	12.24-12.37	734.396	734.397	1.6	yes	715.355	715.355	0.2	
22	11.27	11.17-11.39	11.32	11.22-11.49	940.511	940.514	2.7	yes	921.470	921.469	-0.7	yes
31	11.62	11.57-11.72	11.62	11.51-11.79	940.511	940.515	4.3		921.470	921.472	1.7	
28	11.55	11.45-11.68	11.56	11.41-11.68	1026.512	1026.514	2.2	yes	1007.470	1007.469	-1.4	
35	n. d.	n. d.	11.72	11.68-11.82	1026.512	1026.510	-1.9	yes	1007.470	1007.470	-0.8	
42	11.90	11.82-11.99	11.90	11.83-12.01	1026.512	1026.514	2.4	yes	1007.470	1007.471	1.0	yes
34	11.72	11.66-11.8	11.73	11.69-11.76	1112.512	1112.514	2.2		1093.471	1093.470	-0.5	
41	n. d.	n. d.	11.85	11.75-11.92	1112.512	1112.513	0.9		1093.471	1093.471	0.6	
44	n. d.	n. d.	11.96	11.92-12	1112.512	1112.513	0.9	yes	1093.471	1093.471	0.6	yes
51	12.07	11.96-12.22	12.08	12-12.27	1112.512	1112.514	1.7	yes	1093.471	1093.474	3.0	yes
53	0.00	0-0	0.00	0-0	820.396	0.000			801.355	0.000		
55	12.19	12.06-12.26	12.19	12.11-12.26	820.396	820.396	0.2	yes	801.355	801.356	0.9	yes
62	12.30	12.26-12.38	12.30	12.26-12.38	820.396	820.395	-0.8	yes	801.355	801.358	2.5	
63	n. d.	n. d.	12.42	12.39-12.45	820.396	0.000			801.355	801.351	-4.3	
21	11.25	11.18-11.35	11.26	11.15-11.37	1086.569	1086.574	4.9	yes	1067.528	1067.524	-3.6	yes
27	11.51	11.4-11.66	11.52	11.44-11.65	1172.569	1172.571	1.0	yes	1153.528	1153.530	1.2	yes
6	10.81	10.77-10.86	10.81	10.74-10.88	956.506	956.506	-0.5		937.465	937.467	2.0	
9	10.96	10.86-11.02	10.96	10.88-11.07	956.506	956.505	-0.8		937.465	937.467	2.1	yes

7	n. d.	n. d.	10.81	10.71-10.89	1042.506	1042.506	-0.4		1023.465	1023.464	-0.9	
12	n. d.	n. d.	11.11	11.02-11.31	1042.506	1042.506	-0.1		1023.465	0.000		
14	11.10	11.03-11.18	11.10	11.01-11.13	1042.506	1042.511	4.4		1023.465	1023.465	-0.2	yes
17	11.15	11.14-11.2	n. d.	n. d.	1042.506	1042.506	-0.7		1023.465	1023.467	1.2	
18	11.23	11.2-11.27	n. d.	n. d.	1042.506	0.000		yes	1023.465	1023.463	-1.9	
20	11.31	11.27-11.37	n. d.	n. d.	1042.506	1042.507	0.4		1023.465	1023.465	-0.1	
10	10.96	10.82-11.03	n. d.	n. d.	1128.507	1128.510	3.0	yes	1109.466	1109.466	0.0	yes
23	11.33	11.3-11.37	11.34	11.2-11.37	1128.507	1128.512	4.7		1109.466	1109.466	-0.3	
25	11.42	11.37-11.47	0.00	0-0	1128.507	0.000		yes	1109.466	1109.466	-0.1	yes
30	11.59	11.51-11.66	11.58	11.53-11.64	1214.507	1214.508	0.9		1195.466	1195.466	0.1	yes
33	11.72	11.65-11.76	n. d.	n. d.	1214.507	1214.511	3.8		1195.466	1195.470	4.2	
16	11.13	11.04-11.17	11.14	11.03-11.18	896.449	896.449	0.8		877.407	877.410	2.5	yes
19	11.22	11.17-11.29	11.27	11.18-11.32	896.449	0.000			877.407	877.405	-2.4	yes
40	11.84	11.76-11.89	11.83	11.77-11.89	896.449	896.446	-2.5		877.407	877.410	2.4	yes
1	n. d.	n. d.	10.47	10.39-10.5	1102.564	0.000		yes	1083.523	1083.522	-0.4	yes
2	10.54	10.42-10.62	10.54	10.5-10.63	1102.564	1102.569	4.9		1083.523	1083.523	-0.1	
11	n. d.	n. d.	11.05	10.96-11.13	1102.564	1102.563	-1.3		1083.523	1083.521	-1.8	
4	10.70	10.65-10.74	10.69	10.65-10.74	1188.564	1188.569	4.6		1169.523	1169.523	-0.7	
5	10.79	10.74-10.89	10.79	10.74-10.9	1188.564	1188.569	4.5	yes	1169.523	1169.523	-0.3	yes
8	10.93	10.82-11	10.93	10.82-11.02	1274.565	1274.567	2.2		1255.524	1255.521	-2.4	yes
13	11.09	11.05-11.17	11.09	11.04-11.16	1274.565	1274.566	1.1		1255.524	1255.522	-2.1	
26	11.47	11.4-11.49	11.45	11.31-11.5	982.449	982.451	1.9		963.408	963.393	-15.2	yes
48	12.04	11.93-12.08	12.04	11.9-12.08	982.449	982.523	74.2		963.408	0.000		yes
56	12.24	12.17-12.31	12.20	12.15-12.3	982.449	0.000		yes	963.408	963.411	3.3	
61	n. d.	n. d.	0.00	0-0	982.449	0.000			963.408	0.000		
32	11.64	11.57-11.68	n. d.	n. d.	1068.449	1068.450	1.1		1049.408	1049.408	-0.6	

Table S4. Representative MS/MS spectra of each reported HGL-DTG isomer group. Parent ion mass and measurement error, as well as the ten most abundant fragment m/z with % base peak (bp) intensities (square brackets) are listed. norm. spec. – normalised spectra.

ESI(+) $[M+NH_4]^+$				ESI(-) $[M-H]^-$			
**theor. *m/z***	**MS/MS parent ion m/z**	***m/z* error (ppm)**	**Top 10 MS/MS fragments of norm. spec. (% bp intensity in [ ])**	**theor. *m/z***	**MS/MS parent ion *m/z***	***m/z* error (ppm)**	**Top 10 MS/MS fragments of norm. spec. (% bp intensity in [ ])**
648.3954				629.3543	629.3541	-0.2	59.01 [7.3],71.01 [7.9],85.03 [2.1],89.02 [13.8],101.02 [11.6],113.02 [6.9],119.03 [9.5],161.05 [4.6],467.3 [7.3],629.35 [100]
718.4008				699.3597	699.3601	0.4	59.01 [19.6],85.03 [11.7],161.04 [8.1],163.06 [4.1],413.27 [7.9],449.29 [4],467.3 [14.5],595.35 [21.6],613.36 [47.8],655.37 [100]
734.3957	734.3958	0.1	105.02 [13.9],109.1 [14.4],121.1 [14.8],135.12 [12.1],149.13 [13.8],161.13 [13.9],215.18 [18.2],271.24 [100],289.26 [15.4],734.39 [19.8]	715.3546	715.3550	0.4	92.6 [13.7],101.02 [10.7],178.53 [6.4],245.22 [6.2],380.83 [6.2],392.19 [6.6],611.34 [75.3],629.36 [69.1],671.36 [100],726.25 [7.6]
794.4533	794.4526	-0.7	85.03 [29.2],109.1 [16],129.06 [44],147.07 [36.8],215.18 [15.6],271.24 [100],289.25 [15.5],309.12 [26.8],417.3 [21.8],794.44 [23.3]	775.4122	775.4114	-0.8	59.01 [1.4],89.02 [2.5],101.02 [2.4],113.02 [1.2],119.03 [1.9],467.3 [2.3],611.35 [2.7],629.35 [16.5],775.41 [100],776.41 [1.9]
820.3961	820.3961	0	109.1 [16.7],121.1 [13.4],149.13 [16.2],161.13 [15.3],163.15 [15.9],215.18 [20.3],231.05 [17.3],249.06 [15.3],271.24 [100],820.4 [50.9]	801.3550	801.3549	-0.1	59.01 [24.4],113.02 [7.9],143.03 [4.3],593.33 [23.4],611.35 [72.5],629.35 [26.3],653.35 [41.2],671.36 [50.4],713.38 [100],801.36 [15.4]
880.4537	880.4540	0.3	85.03 [18.1],215.18 [17.5],231.05 [21.1],249.06 [17.9],271.24 [100],289.25 [16.4],359.1 [28.4],377.11 [17.5],395.12 [47.2],880.45 [30.2]	861.4126	861.4145	1.9	89.02 [0.8],467.3 [1.4],611.35 [4.7],629.36 [5.3],757.4 [26.2],775.41 [22.3],817.43 [100],818.42 [2.5],861.42 [24.1],862.41 [1]
896.4486				877.4075	877.4079	0.4	175.56 [1.5],542.39 [1.5],576.81 [1.7],611.35 [1.5],629.35 [4.1],773.39 [30.4],791.4 [20.5],833.42 [100],834.43 [2.6],877.4 [19.8]
940.5112	940.5109	-0.3	85.03 [19.4],129.06 [31.6],147.07 [28.2],271.24 [100],273.1 [15.4],293.12 [63.2],309.12 [29.6],417.3 [36.6],455.17 [20.6],940.5 [40.2]	921.4701	921.4695	-0.6	163.06 [0.4],467.3 [0.2],613.36 [0.3],629.36 [0.3],757.4 [0.7],759.42 [0.8],775.41 [5.4],921.17 [0.5],921.46 [100],922.47 [3.5]
956.5061				937.4650	937.4653	0.3	112.98 [1.3],180.97 [0.6],324.15 [0.4],549.35 [0.4],629.35 [0.9],775.41 [1.9],791.4 [1.6],893.39 [0.7],937.48 [100],938.46 [3.9]
966.4540	966.4546	0.6	109.1 [11.8],215.18 [12.8],231.05 [16.9],249.06 [16.5],271.24 [100],289.25 [11.9],359.1 [14.9],395.12 [31.3],417.3 [21.2],966.46 [88.7]	947.4129	947.4135	0.6	611.35 [5],739.39 [13.3],757.4 [21.1],775.41 [7.9],799.4 [36.2],817.43 [31.3],859.44 [100],903.43 [5.6],947.22 [3.5],947.41 [53]
982.4490	982.5211	72.1	85.03 [13.7],109.1 [9.6],129.05 [26.2],147.07 [18.9],205.07 [9.2],271.24 [66.2],275.11 [9.8],293.12 [50.3],417.3 [30.4],982.51 [100]	963.4079	963.4076	-0.3	815.4 [19.2],831.33 [9.7],833.43 [20.4],873.35 [21.7],875.44 [70.6],917.35 [32.2],918.35 [8.4],919.34 [11.7],962.34 [58.3],963.35 [100]
1026.5116	1026.5104	-1.2	85.03 [10.9],129.05 [15.8],147.06 [10.7],271.24 [56.2],293.12 [44.6],359.1 [16.6],377.11 [11],395.12 [28.6],417.3 [21.5],1026.51 [100]	1007.4705	1007.4709	0.4	757.4 [1.6],775.41 [2.4],817.43 [1.5],903.45 [9.5],904.46 [0.9],921.46 [8.3],963.49 [100],964.48 [4.4],1007.46 [46.7],1008.46 [2.9]
1042.5065	1042.5061	-0.4	145.05 [8.6],215.18 [7.6],271.24 [58.2],289.25 [11],309.12 [20.4],359.1 [9.6],395.12 [18.4],411.11 [7.6],417.3 [10.7],1042.51 [100]	1023.4654	1023.4667	1.3	412.19 [0.5],486.79 [0.5],773.39 [0.9],791.4 [1.1],919.45 [14.7],937.48 [7.9],979.49 [100],980.48 [4],1023.48 [43.9],1024.48 [1.5]

1068.4494				1049.4083			
1086.5691	1086.5682	-0.9	129.05 [17.6],147.06 [15.5],271.24 [59.5],293.12 [78],309.12 [20.8],399.29 [14.2],417.3 [36.3],439.18 [13.3],455.18 [12.5],1086.57 [100]	1067.5280	1067.5269	-1.1	92.6 [0.4],228.72 [0.2],300.96 [0.2],662.65 [0.2],775.41 [0.2],903.45 [0.3],921.46 [1.9],932.95 [0.2],1067.52 [100],1068.55 [4.3]
1102.5640	1102.5637	-0.3	129.05 [8],271.24 [18.8],273.1 [11.1],293.12 [25.1],309.12 [25.5],417.3 [10.2],455.18 [13.3],471.17 [8],617.23 [9.7],1102.58 [100]	1083.5229	1083.5231	0.2	112.98 [0.1],308.12 [0.1],555.49 [0.1],775.41 [0.1],919.48 [0.1],921.46 [0.4],937.45 [0.9],1083.14 [0.6],1083.51 [100],1084.53 [4.9]
1112.5120	1112.5114	-0.6	215.18 [5.9],231.05 [6.1],271.24 [36.2],293.12 [11.3],359.1 [8.1],377.11 [6.3],395.12 [16.4],399.29 [5.3],417.3 [11.7],1112.5 [100]	1093.4709	1093.4706	-0.3	817.43 [2.2],885.44 [1.7],903.45 [3.2],945.48 [14],963.49 [12.4],1005.49 [100],1006.49 [5.5],1049.49 [8.9],1093.46 [71.7],1094.49 [4.9]
1128.5069	1128.5065	-0.4	231.05 [10.2],249.06 [9.5],271.24 [48],359.1 [9.3],393.1 [6.9],395.12 [20.3],411.11 [9.8],417.3 [9.2],557.17 [9.5],1128.51 [100]	1109.4658	1109.4662	0.4	901.45 [1.1],919.45 [1.7],937.45 [1.2],961.45 [10.3],979.49 [11.4],1021.5 [100],1022.48 [2.9],1065.47 [6.7],1109.48 [60.2],1110.46 [1.7]
1172.5695	1172.5693	-0.2	129.05 [6],271.24 [24.7],293.12 [33.6],359.1 [5.4],377.11 [5],395.12 [15.4],399.29 [6.2],417.3 [16.8],541.18 [6.1],1172.59 [100]	1153.5284	1153.5294	1.0	903.45 [0.5],921.45 [1.1],963.49 [0.7],1049.52 [5.9],1050.53 [0.4],1067.52 [3.7],1109.55 [100],1110.52 [5.5],1153.54 [70.4],1154.51 [3.7]
1188.5644	1188.5641	-0.3	129.06 [5.6],271.24 [26.3],273.1 [8],293.12 [16.3],309.12 [19.8],417.3 [8.6],455.18 [7],471.17 [10.6],617.23 [6.1],1188.55 [100]	1169.5233	1169.5238	0.5	226.45 [0.6],261.44 [0.5],612.01 [0.5],1065.51 [3.6],1083.51 [3.4],1125.53 [100],1126.51 [2.7],1169.54 [63.3],1170.53 [1.8],1182.7 [0.6]
1214.5073				1195.4662	1195.4668	0.6	943.44 [1.8],961.48 [3.6],1003.47 [10.2],1021.49 [13.7],1063.5 [68],1064.52 [2.9],1107.48 [2.1],1151.46 [12.8],1195.45 [100],1196.47 [3.1]
1274.5648				1255.5237	1255.5237	0.0	166.46 [0.8],585.26 [0.9],1107.52 [4.7],1125.53 [4.3],1167.53 [72],1168.55 [5.5],1169.54 [0.9],1211.53 [12.3],1255.53 [100],1256.53 [5.6]
